# Supplementary material for: The Gram-positive bacterium Romboutsia ilealis harbors a polysaccharide synthase that can produce (1,3;1,4)-β-d-glucans
Source: Nat Commun. 2023 Jul 27;14:4526. doi: 10.1038/s41467-023-40214-z (PMC10374906; doi:10.1038/s41467-023-40214-z)
Supplement: Supplementary file 1 — Supplementary Information [file 41467_2023_40214_MOESM1_ESM.pdf]

Supplementary Information

The Gram-positive bacterium *Romboutsia ilealis* harbors a polysaccharide synthase that can produce (1,3;1,4)-β-D-glucans

Shu-Chieh Chang[1,2], Mu-Rong Kao[1,2], Rebecka Karmakar Saldivar[1,2], Sara M. Díaz-Moreno[1], Xiaohui Xing[3], Valentina Furlanetto[4], Johannes Yayo[4], Christina Divne[4], Francisco Vilaplana[1], D. Wade Abbott[3], Yves S. Y. Hsieh[1,2]

Author Information

Affiliations

[1]Division of Glycoscience, Department of Chemistry, School of Engineering Sciences in Chemistry, Biotechnology and Health, KTH Royal Institute of Technology, AlbaNova University Centre, Stockholm, SE10691, Sweden

[2]School of Pharmacy, College of Pharmacy, Taipei Medical University, 250 Wuxing Street, Taipei 11031, Taiwan

[3]Lethbridge Research and Development Centre, Agriculture and Agri-Food Canada, Lethbridge, Alberta T1J 4B1, Canada

[4]Department of Industrial Biotechnology, School of Engineering Sciences in Chemistry, Biotechnology and Health, KTH Royal Institute of Technology, AlbaNova University Centre, Stockholm, SE10691, Sweden

Supplementary Information
Figures and Tables

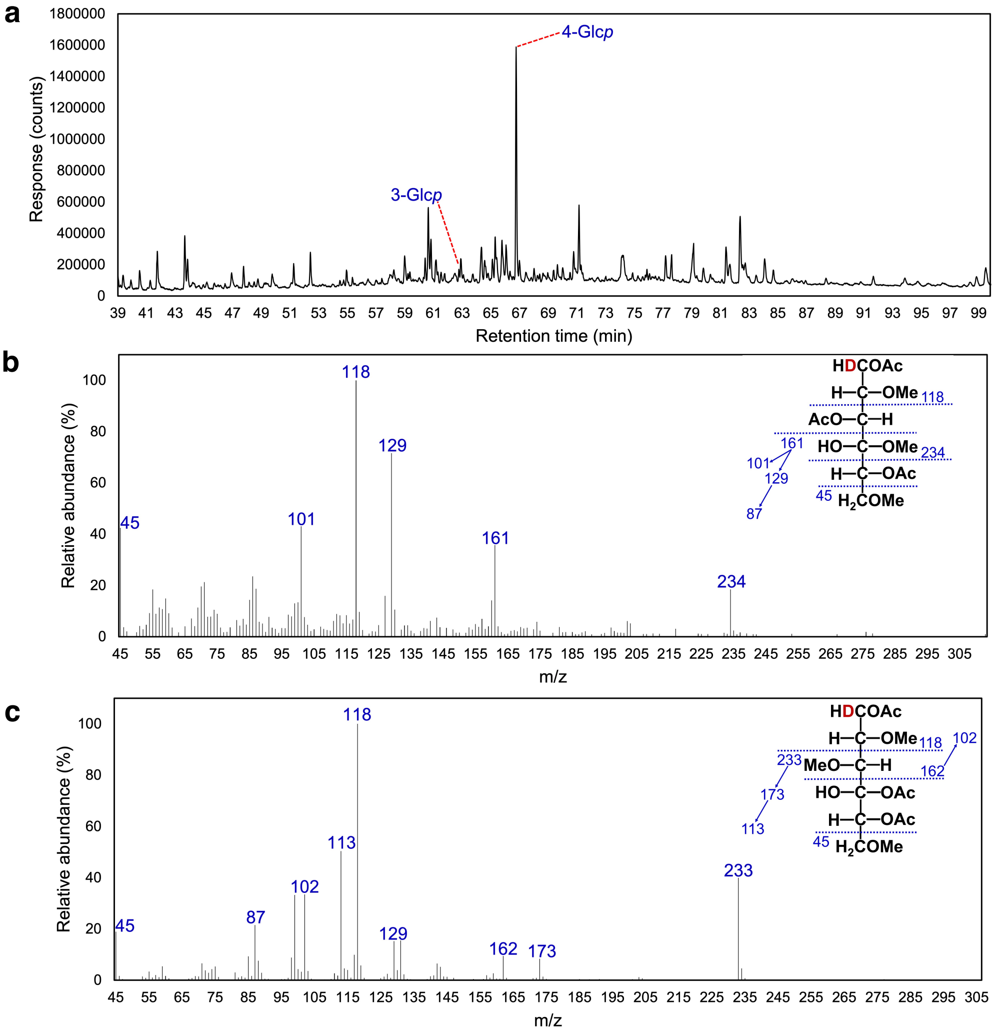

Supplementary Figure 1. Linkage analysis of *R. ilealis* CRIB^{T} EPS.

Partially methylated alditol acetates (PMAAs) resulting from linkage analysis of *R. ilealis* CRIB^{T} EPS were analyzed by GC-MS on a SP2380 capillary column with a stabilized poly (90% biscyanopropyl/10% cyanopropylphenyl siloxane) phase that completely suppresses signals from amino sugar linkages. **a.** The total ion current (TIC) chromatogram, with the peaks for the PMAAs corresponding to 3-linked glucopyranose (3-Glc*p*) and 4-linked glucopyranose (4-Glc*p*) marked. **b.** and **c.** The EI-MS spectra of the PMAAs corresponding to 3-Glc*p* and 4-Glc*p*, respectively. The structures of these PMAAs are shown together with their fragmentation patterns. Two separate experiments were conducted.

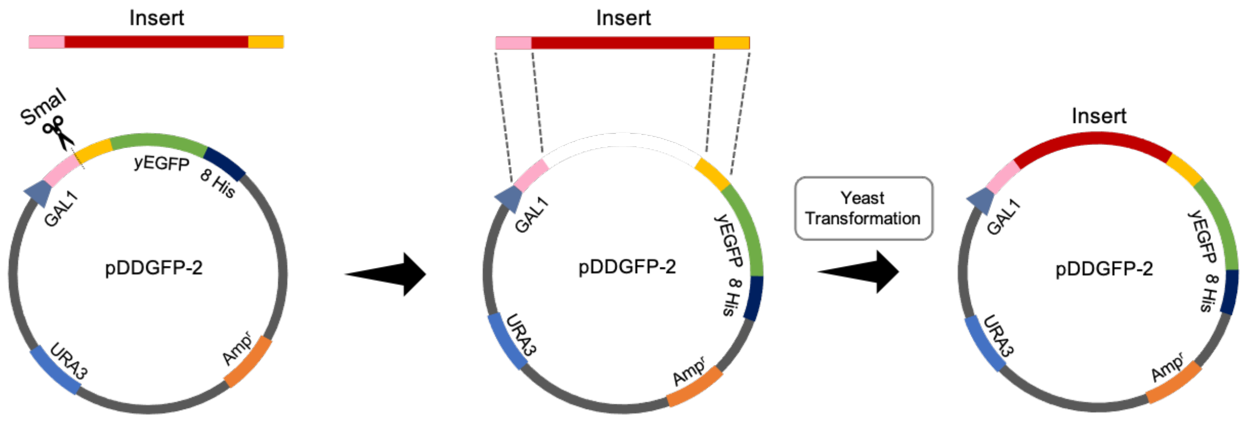

Supplementary Figure 2. Cloning by homologous recombination into the *Saccharomyces cerevisiae* GFP-fusion vector.

The 2µ GFP-fusion pDDGFP-2 vector was linearized with SmaI restriction enzyme (Thermo Scientific). The gene of interest (insert) was amplified with an overhanging sequence that is required for homologous recombination and, together with the linear vector, was transformed into *Saccharomyces cerevisiae* LoGSA[31]. *GAL1*, galactose inducible promoter; yEGFP, yeast-enhanced green fluorescent protein.

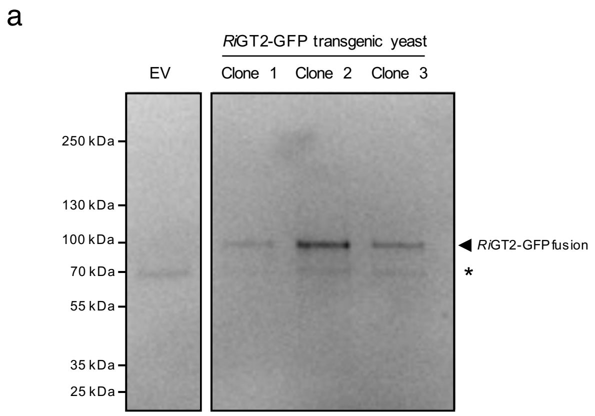

Supplementary Figure 3. Clone screening and enrichment of the *Ri*GT2-GFP fusion proteins.

a. The clones were cultured on a small scale (10 mL), and a crude membrane preparation was isolated and separated by SDS-PAGE. The expressed *Ri*GT2-GFP-His8 fusion protein with a molecular weight of 102.7 kDa is visible at the 100 kDa mark (clone 1 to 3), whereas the *Ri*GT2-GFP-His_8 is absent in the empty vector (EV). Representative of 3 independent experiments. The asterisk shows the position of endogenous fluorescent 'background' protein. **b.** The expressed *Ri*GT2-GFP-His8 fusion proteins were enriched from a 10 L culture using nickel-charged IMAC resin (Millipore, USA). The left panel shows an SDS-PAGE gel stained with Coomassie Brilliant Blue of the enriched proteins, and the right panel shows an SDS-PAGE where *Ri*GT2-GFP-His_8 has been visualized by in-gel fluorescence. The *Ri*GT2-GFP-His8 fusion protein is indicated by an arrow in both panels. A gel band at this position (~100 kDa) was removed for tryptic digestion and analysis by mass spectrometry (MS/MS). Representative of 3 independent experiments.

Compute pI/Mw

Theoretical pI/Mw (average) for the user-entered sequence:

```
        10         20         30         40         50         60
MYALIMVITL LLSYIVSKQK VEYRKILIFI NAVVCCIYII WRITVIPIHS GIISFLLGIT

        70         80         90        100        110        120
LFLAEALGLI SFLNFKYLFT KKYKLELKTL DDFQYGNIPY VDVLICTYNE PLYLLEKTIA

       130        140        150        160        170        180
ASTNLDYPTH KFKIHVCDDG RRDSLKLLCK KYNVNYISRD NNEGAKAGNI NNALKYLKGD

       190        200        210        220        230        240
LFAVLDADMI PKKEFLSRTV GYFTNENLAF VQVPQVYYNK DTYQYNLMKN IPNEQDFFMR

       250        260        270        280        290        300
DIQEARASIN AVLHVGTNAL FKREYVNEIG GYPTCSITED MAVGMLLQSR GYDSVFINEE

       310        320        330        340        350        360
LVLGLSATTF TELVKQRDRW CRGNIQVLKH FNPIFTKGLT LPQKIAYFDG GVYWFSNLQK

       370        380        390        400        410        420
IVFILFPIIY LLTRKLIIDS SILTLLNMYI PFILGQILIF NTLSPGNRKL TWAHFYEIAM

       430        440        450        460        470        480
APHLTLSILK EMLFLKTKFN VTLKEIQQDK KQFQFRVALP HIVIVIVTII AWIVSTRLLI

       490        500        510        520        530        540
EKNIHVQAYL LNMIWSIYNF IGAIICIKVS YQKPIFRTSE RININEDITV ECDYKQNKFK

       550        560        570        580        590        600
AKILNLSEKG IGLKLNEELD LQCEETIKLD LKGSIFICKI SRINKDLLGL SFNKVTPYQM

       610        620        630        640        650        660
KLIMSIFTEN MQPYYKIAKS QEYIVNKKEV AEVAMVSGEN LYFQGQFSKG EELFTGVVPI

       670        680        690        700        710        720
LVELDGDVNG HKFSVSGEGE GDATYGKLTL KFICTTGKLP VPWPTLVTTF GYGVQCFARY

       730        740        750        760        770        780
PDHMKQHDFF KSAMPEGYVQ ERTIFFKDDG NYKTRAEVKF EGDTLVNRIE LKGIDFKEDG

       790        800        810        820        830        840
NILGHKLEYN YNSHNVYIMA DKQKNGIKVN FKIRHNIEDG SVQLADHYQQ NTPIGDGPVL

       850        860        870        880        890
LPDNHYLSTQ SALSKDPNEK RDHMVLLEFV TAAGITHGMD ELYKHHHHHH HH
```

Theoretical pI/Mw: 8.52 / 102741.58

Supplementary Figure 4. Amino-acid sequence of the *Ri*GT2-TEV-GFP-His$_8$ construct.

The fusion protein consists of *Ri*GT2, a TEV protease cleavage site (red), eGFP (green), and a *C*-terminal polyHis-tag (blue). The predicted molecular weight is 102.7 kDa using the ExPASy Compute pI/Mw tool (https://web.expasy.org/compute_pi/). TEV, Tobacco Etch Virus; eGFP, enhanced green fluorescent protein.

Protein sequence coverage: 37%

Matched peptides shown in ***bold red***.

**1**	MYALIMVITL	LLSYIVSKQK	VEYRKILIFI	NAVVCCIYII	WRITVIPIHS
**51**	GIISFLLGIT	LFLAEALGLI	SFLNFKYLFT	KKYKLELKTL	DDFQYGNIPY
**101**	VDVLICTYNE	PLYLLEK**TIA**	**ASTNLDYPTH**	**K**FKIHVCDDG	RRDSLKLLCK
**151**	**KYNVNYISR**D	NNEGAK**AGNI**	**NNALKYLKGD**	**LFAVLDADMI**	**PK**KEFLSR**TV**
**201**	**GYFTNENLAF**	**VQVPQVYYNK**	**DTYQYNLMKN**	**IPNEQDFFMR**	DIQEARASIN
**251**	AVLHVGTNAL	FKREYVNEIG	GYPTCSITED	MAVGMLLQSR	**GYDSVFINEE**
**301**	**LVLGLSATTF**	**TELVK**QRDRW	CRGNIQVLK**H**	**FNPIFTK**GLT	LPQK**IAYFDG**
**351**	**GVYWFSNLQK**	IVFILFPIIY	LLTRKLIIDS	SILTLLNMYI	PFILGQILIF
**401**	NTLSPGNRKL	TWAHFYEIAM	APHLTLSILK	EMLFLK**TKFN**	**VTLK**EIQQDK
**451**	KQFQFRVALP	HIVIVIVTII	AWIVSTRLLI	EKNIHVQAYL	LNMIWSIYNF
**501**	IGAIICIKVS	YQKPIFRTSE	RININEDITV	ECDYKQNKFK	AK**ILNLSEK**G
**551**	IGLKLNEELD	LQCEETIKLD	LKGSIFICKI	SR**INKDLLGL**	**SFNK**VTPYQM
**601**	K**LIMSIFTEN**	**MQPYYK**IAK**S**	**QEYIVNKKEV**	**AEVAMVSGEN**	**LYFQGQFSK**G
**651**	EELFTGVVPI	LVELDGDVNG	HK**FSVSGEGE**	**GDATYGK**LTL	KFICTTGKLP
**701**	VPWPTLVTTF	GYGVQCFARY	PDHMKQHDFF	K**SAMPEGYVQ**	**ERTIFFKDDG**
**751**	**NYK**TR**AEVKF**	**EGDTLVNR**IE	LK**GIDFKEDG**	**NILGHKLEYN**	**YNSHNVYIMA**
**801**	**DK**QKNGIKVN	FK**IRHNIEDG**	**SVQLADHYQQ**	**NTPIGDGPVL**	**LPDNHYLSTQ**
**851**	**SALSK**DPNEK	RDHMVLLEFV	TAAGITHGMD	ELYKHHHHHH	HH

Supplementary Figure 5. Tryptic peptides matched to the *Ri*GT2-GFP-His$_8$ fusion protein sequence.

The peptides released from the tryptic in-gel digestion were analyzed by LC-MS. The peptides were matched to the *Ri*GT2-GFP-His$_8$ fusion-protein sequence with 37% coverage.

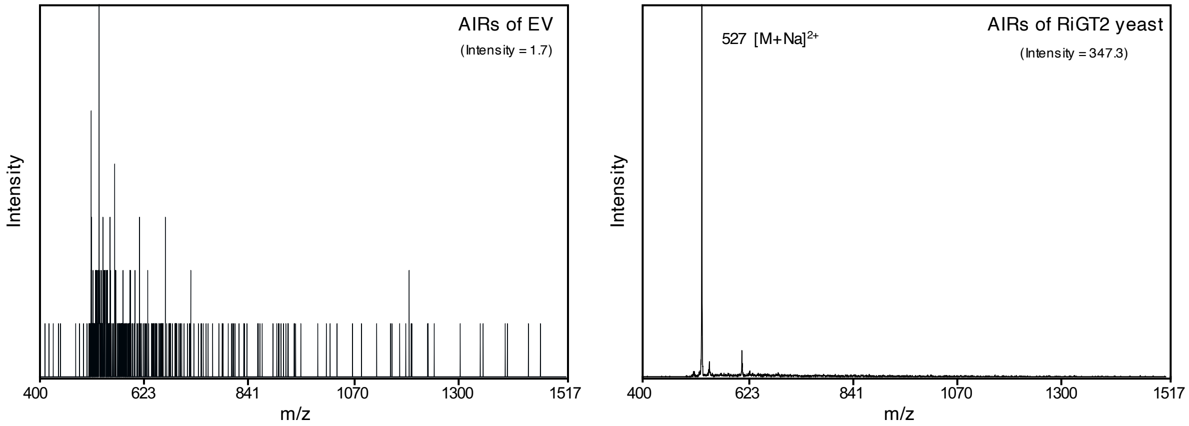

Supplementary Figure 6. MALDI-TOF MS spectra of lichenase hydrolysates of *RiGT2* LoGSA and EV LoGSA.

The molecular ion peak with an *m/z* of 527 corresponding to that of the DP3 oligosaccharide of (1,3;1,4)-β-D-glucans was detected in the lichenase hydrolysate of the AIR of *Ri*GT2 LoGSA, but no oligosaccharides in the 400-1500 Da range from (1,3;1,4)-β-D-glucans were detected in the lichenase hydrolysate of the EV AIR.

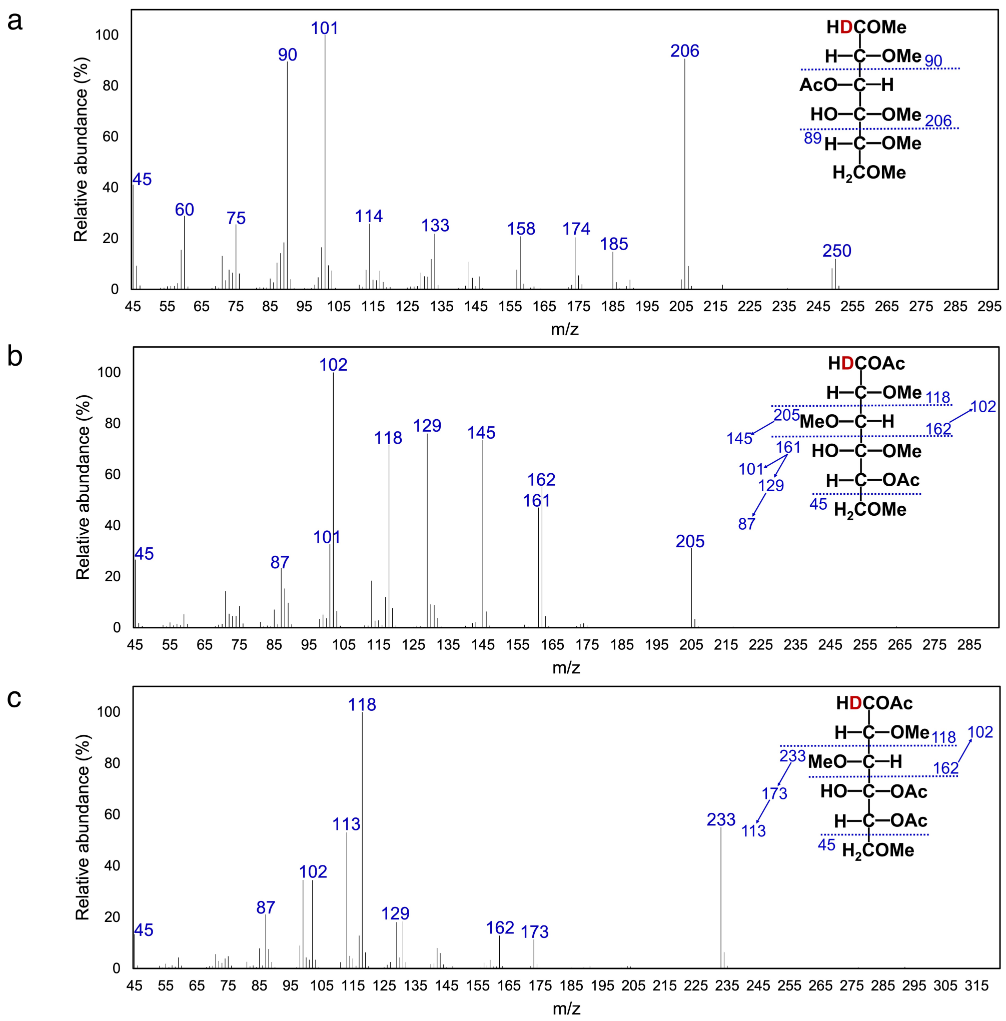

Supplementary Figure 7. Mass spectra of PMAAs from the linkage analysis.

Mass spectra of the partially methylated alditol acetates (PMAAs) obtained in the linkage analysis of the DP3 oligosaccharide purified from lichenase hydrolysates of the AIR obtained from *RiGT2* LoGSA. The DP3 oligosaccharide was reduced by $NaBD_4$ and examined by linkage analysis. The PMAAs derivatives obtained were analyzed by GC-MS. The resulting three peaks correspond to 3-glucitol, t-Glc*p* and 4-Glc*p* (see Figure 4). Panels **a**, **b** and **c** show the EI-MS spectra and fragmentation patterns of the PMAAs corresponding to 3-glucitol, t-Glc*p* and 4-Glc*p,* respectively. Two separate experiments were conducted.

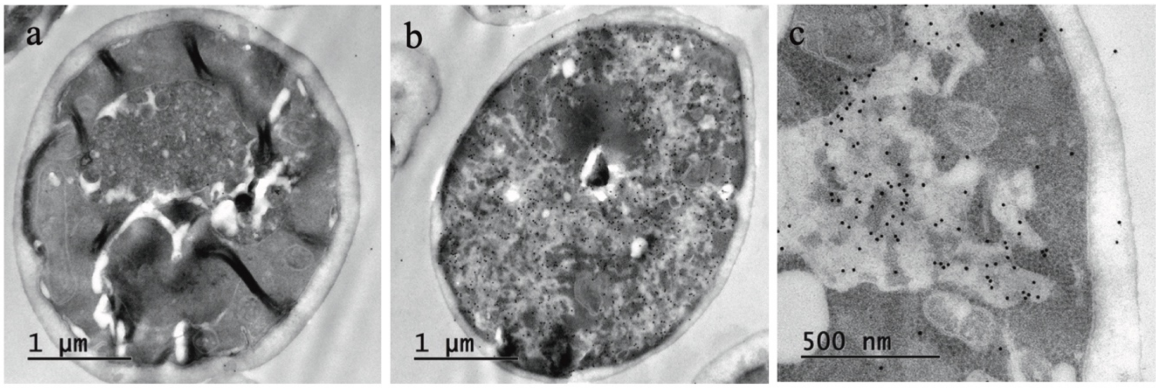

Supplementary Figure 8. Indirect immunogold microscopy of LoGSA yeast cells.

Indirect immunogold microscopy was carried out using transmission electron microscopy (TEM) on ultrathin sections of *RiGT2* LoGSA yeast cells. A primary monoclonal antibody specific for (1,3;1,4)-β-D-glucans (BS400-3) and a colloidal-gold labeled secondary antibody were used.
a. Non-induced *RiGT2* LoGSA cells. **b** and **c.** Induced *RiGT2* LoGSA cells. Compared with the non-induced *RiGT2* LoGSA cells, the induced *RiGT2* LoGSA cells showed intense labeling over the cytoplasm indicating that (1,3;1,4)-β-D-glucans were present in the *Ri*GT2 LoGSA yeast cells. Representative of 3 independent experiments.

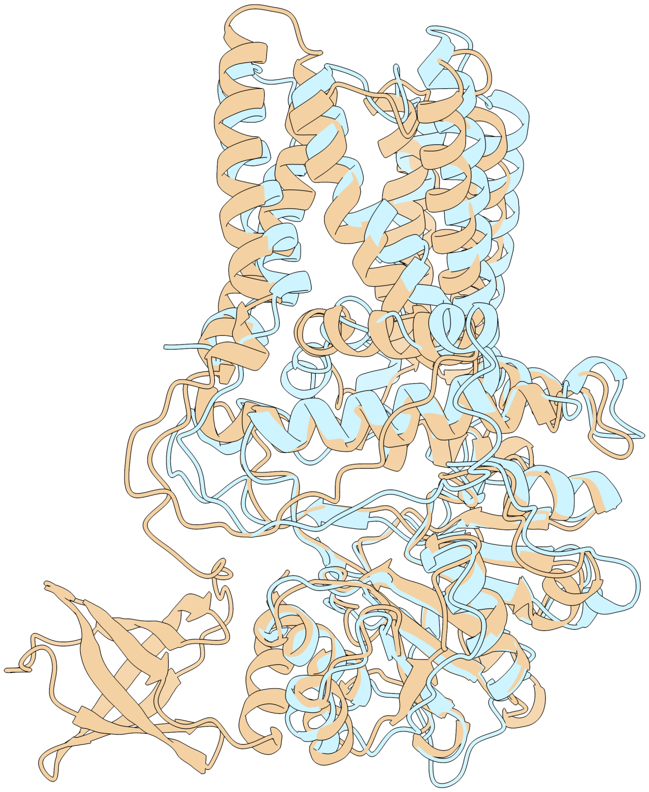

Supplementary Figure 9. Comparison of the *Ri*GT2 models generated by AlphaFold2 and SWISS-MODEL.

Comparison of the two theoretical *Ri*GT2 models generated by AlphaFold2 (orange) and SWISS-MODEL (blue). The model built by SWISS-MODEL lacks the PilZ domain since the *Hv*CslF6 template does not have a PilZ domain. The figure was prepared by UCSF ChimeraX[76].

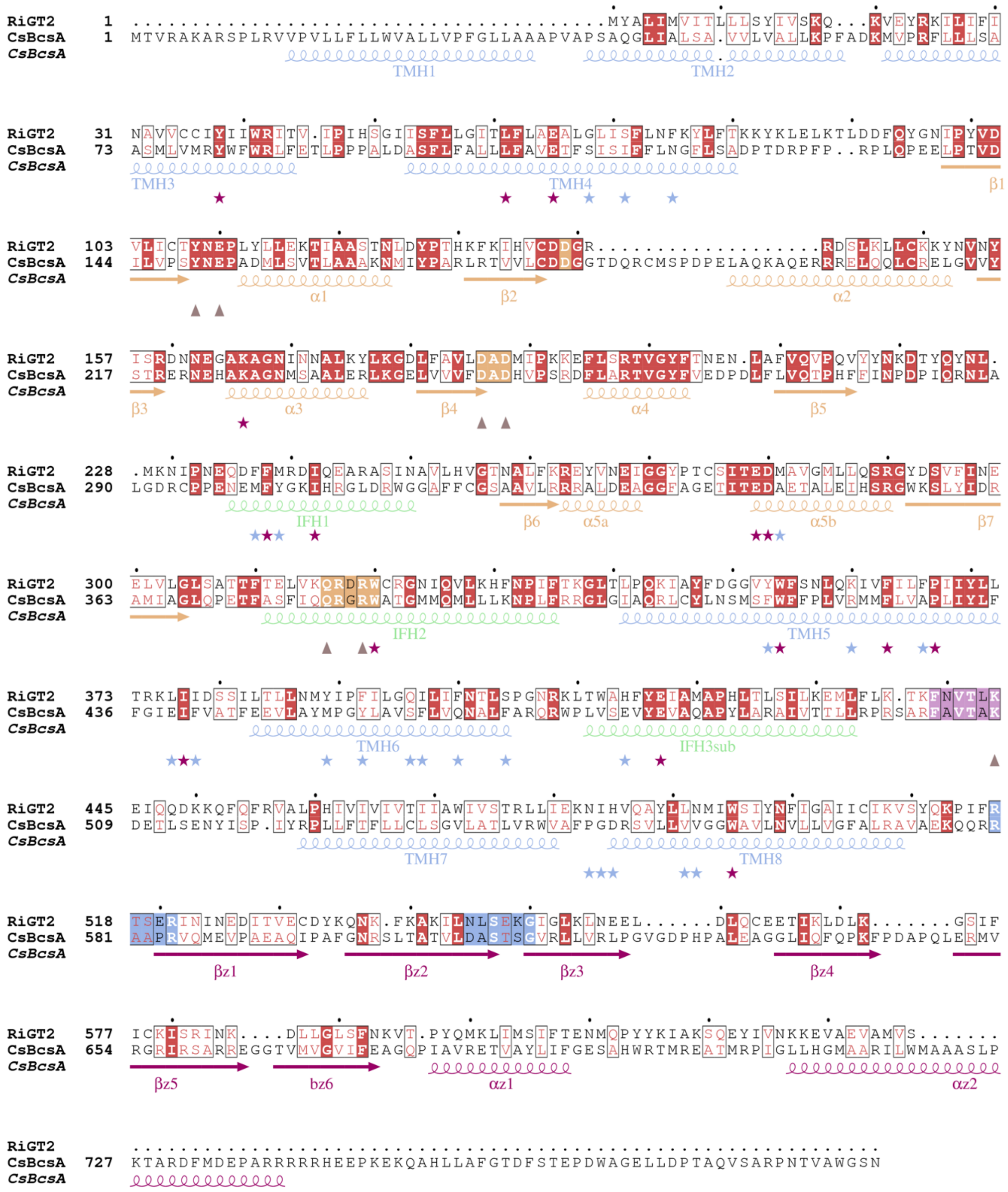

Supplementary Figure 10. Structure-optimized sequence alignment for *Ri*GT2 and *Cs*BcsA.

The secondary-structure elements for *Cs*BcsA are indicated below the sequence. Transmembrane helices (TMH1-8) are in blue, interface α-helices (IFH1-3) in green, α-helices and β-strands belonging to the glycosyltransferase (GT) domain are in orange, and α-helices and β-strands in the PilZ domain are in purple. Conserved residues are highlighted by red boxes. The [D,D,D,Q(Q/R)XRW] motifs in the GT domain are shown as orange filled boxes, and the gating-loop motif [FXVTXK] in purple boxes. The motifs in the PilZ domain, [RXXXR] and

[D/NXSXXG] are shown with blue boxes. Residues in *Cs*BcsA that contact the UDP-Glc donor are indicated by brown up-triangles. Residues that interact with a cellulose chain in *Cs*BcsA and are conserved in *Ri*GT2 are indicated by purple starts, and those cellulose-interacting residues that are not conserved in *Ri*GT2 as blue stars.

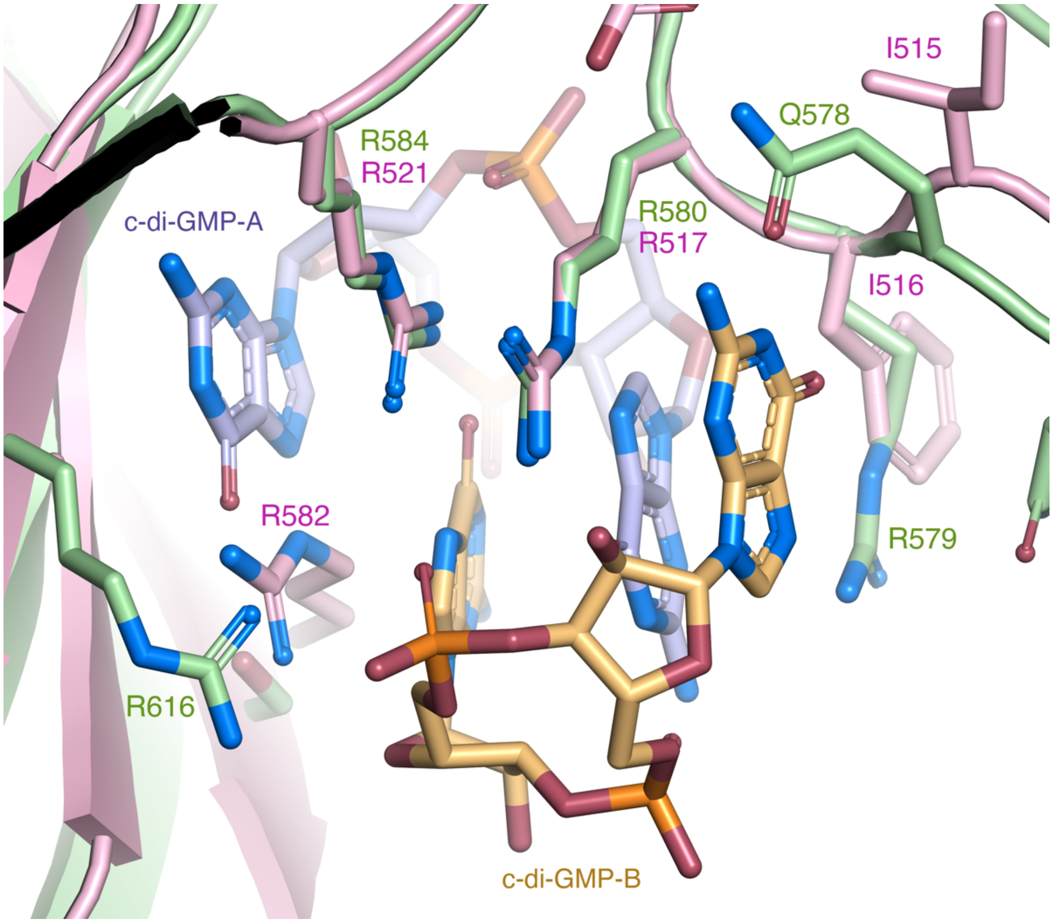

Supplementary Figure 11. Comparison of the c-di-GMP-binding RxxxR motif in *Cs*BcsA and *Ri*GT2.

Superposition of the c-di-GMP-binding region of the PilZ domain in *Cs*BcsA (green; PDB 4P00 [https://www.rcsb.org/structure/4P00]) and the *Ri*GT2$_A$ model (pink). The PilZ domain in *Cs*BcsA binds a c-di-GMP dimer and represents the open state. The c-di-GMP monomer A is colored light blue and monomer B is orange. The arginine pair of the RxxxR motif is conserved in both proteins (Arg580/Arg584 in *Cs*BcsA and Arg517/Arg521 in *Ri*GT2). The positions occupied by Arg579 and Arg616 in *Cs*BcsA are not conserved among PilZ domains, and in *Ri*GT2, these residues are Phe516 and Gly552, respectively. The role of Arg579 in binding c-di-GMP in *Cs*BcsA could however be performed by Arg582 loacted on a nearby β-strand in *Ri*GT2. The figure was prepared by PyMOL (Schrödinger, L. & DeLano, W., 2020. PyMOL, Available at: http://www.pymol.org/pymol).

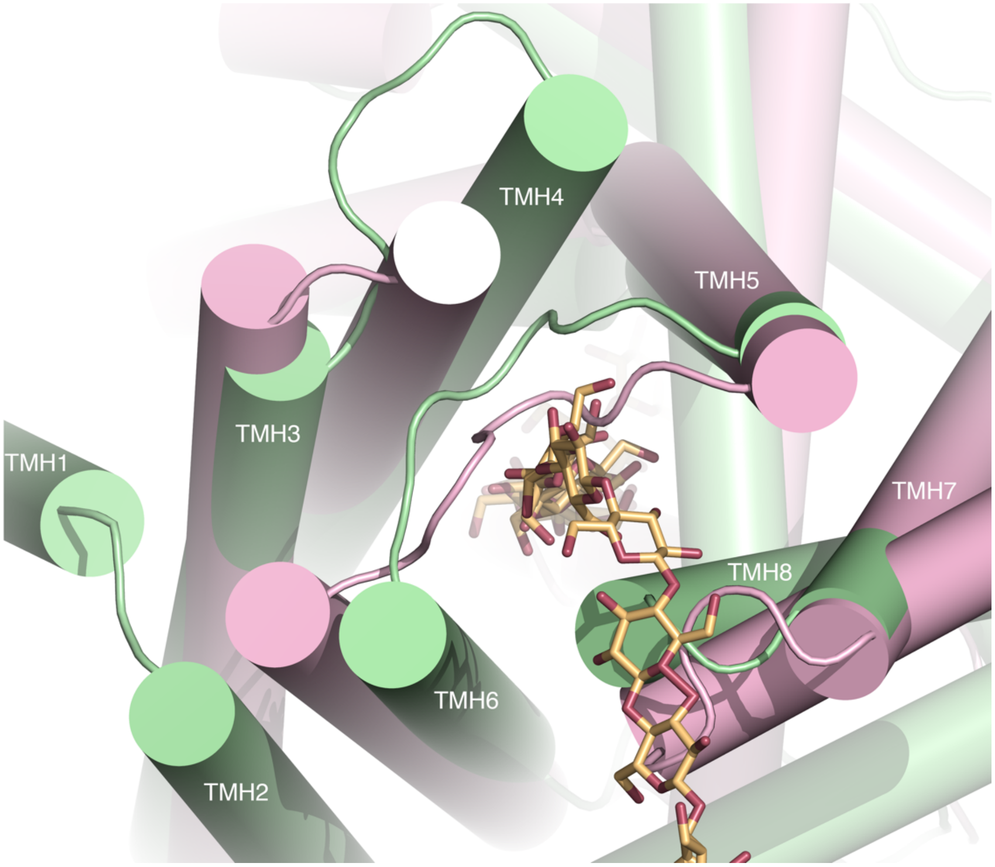

Supplementary Figure 12. Positions of the TMHs in *Cs*BcsA and *Hv*CslF6 that form the channel.

Superposition of the TMHs in *Cs*BcsA (green) with its bound cellulose chain (PDB 4P02 [https://www.rcsb.org/structure/4P02]) and *Hv*CslF6 (pink; PDB 8DQK [https://www.rcsb.org/structure/8DQK]). *Hv*CslF6 lacks TMH1 and TMH2. The TMHs in *Hv*CslF6 corresponding to TMH3, TMH4 and TMH8 in *Cs*BcsA show displacements that create a larger channel. The figure was prepared by PyMOL (Schrödinger, L. & DeLano, W., 2020. PyMOL, Available at: http://www.pymol.org/pymol).

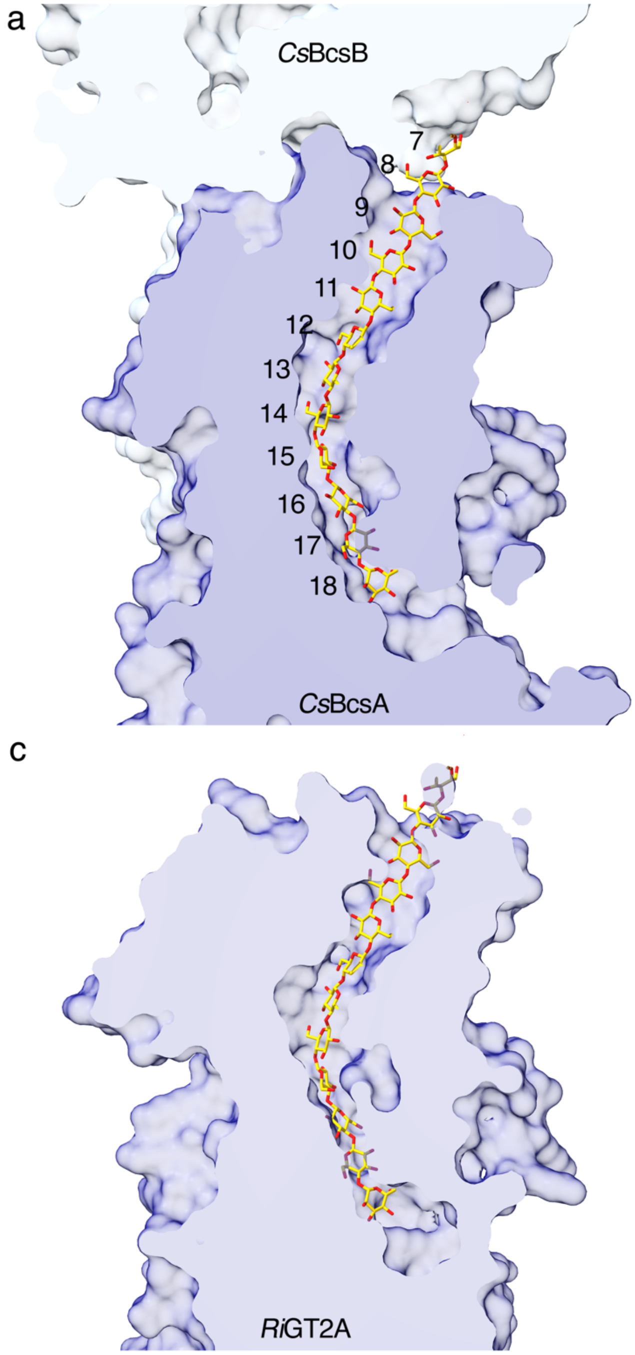

Supplementary Figure 13. Comparison of the channels in *Cs*BcsA, *Hv*CslF6 and the theoretical *Ri*GT2 models.

a. *Cs*BcsA-*Cs*BcsB complex (PDB 4HG6); **b.** *Hv*CslF6 (PDB 8DQK); **c.** *Ri*GT2 model generated by AlphaFold2; and **d.** *Ri*GT2 model generated by SWISS-MODEL using *Hv*CslF6 as template. The cellulose chain in the *Cs*BcsA-*Cs*BcsB complex has been overlaid on the models in panels b-d. The template bias from *Cs*BcsA of the AlphaFold2 model of *Ri*GT2 is visible by comparing panels **a** and **c**. Similarly, the template bias from *Hv*CslF6 in the *Ri*GT2S model generated by

SWISS-MODEL is also obvious from comparing panels b and d. The figure was prepared by UCSF ChimeraX[76].

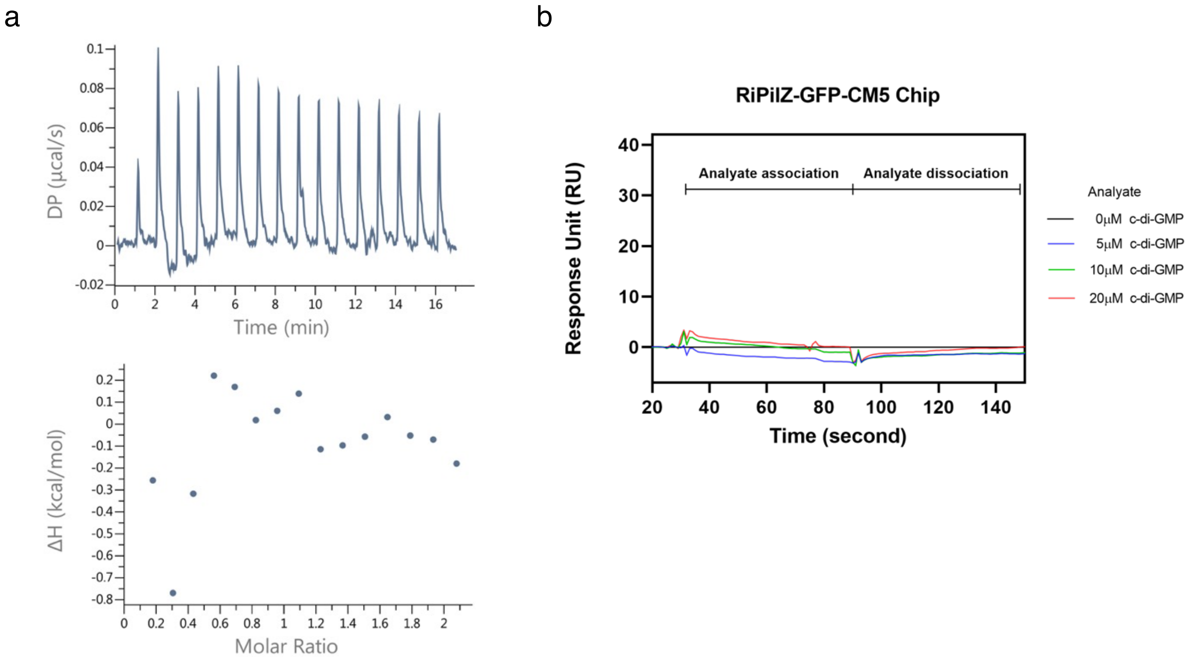

Supplementary Figure 14. Analysis of binding of c-di-GMP to the *Ri*PilZ-GFP fusion protein by ITC and SPR.

No binding of c-di-GMP to the *Ri*PilZ-GFP fusion protein was detected by ITC or SPR. **a.** ITC data showing the thermodynamic parameters of protein-ligand interactions. The top panel shows the calorimetric titration, and the bottom panel shows the derived binding isotherm plotted against the molar ratio of the titrant. **b.** SPR analysis of possible binding of c-di-GMP to *Ri*PilZ-GFP fusion protein. The sensorgrams are representative of experiments that were carried out using four different concentrations of the analyte.

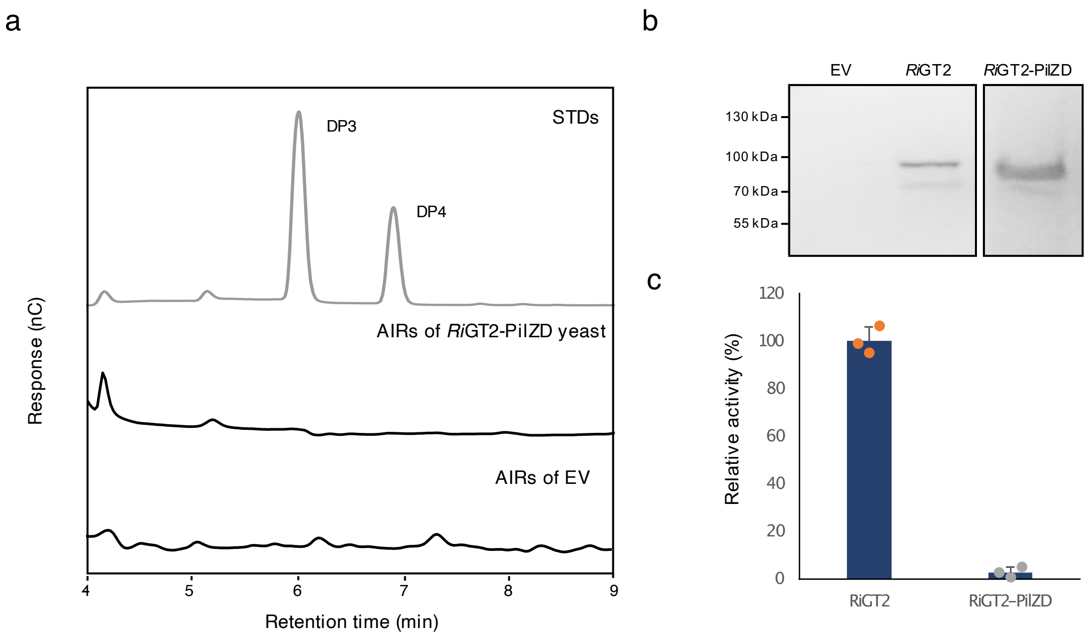

Supplementary Figure 15. Activity analysis of the *Ri*GT2-PilZD mutant.

The *Ri*GT2-PilZD mutant corresponds to *Ri*GT2 with the PilZ deleted and did not synthesize (1,3;1,4)-β-D-glucans either *in vivo* or *in vitro*. **a.** The lichenase hydrolysate of the AIR from *Ri*GT2-PilZD LoGSA was analysed by HPAEC-PAD and compared with the hydrolysate of the AIR from the EV transgenic yeast. No peaks occur with the retention times of the pure DP3 or DP4 standard oligosaccharides from (1,3;1,4)-β-D-glucans. **b**. The clone screening by in-gel fluorescence. The predicted molecular weight of *Ri*GT2-PilZD with C-terminal GFP fusion is 85 kDa. Representative of 3 independent experiments. **c**. The *in vitro* (1,3;1,4)-β-D-glucan synthase activities of the *Ri*GT2 and *Ri*GT2-PilZD microsomal fractions were measured using a radiometric assay. The *Ri*GT2-PilZD microsomal fraction showed no synthase activity calculated relative to the activity of the *Ri*GT2. Data are presented as mean values ± SD. Each data point represents a independent experiment. The error bars indicate the standard deviation (SD) calculated from three replicates (n = 3).

a.

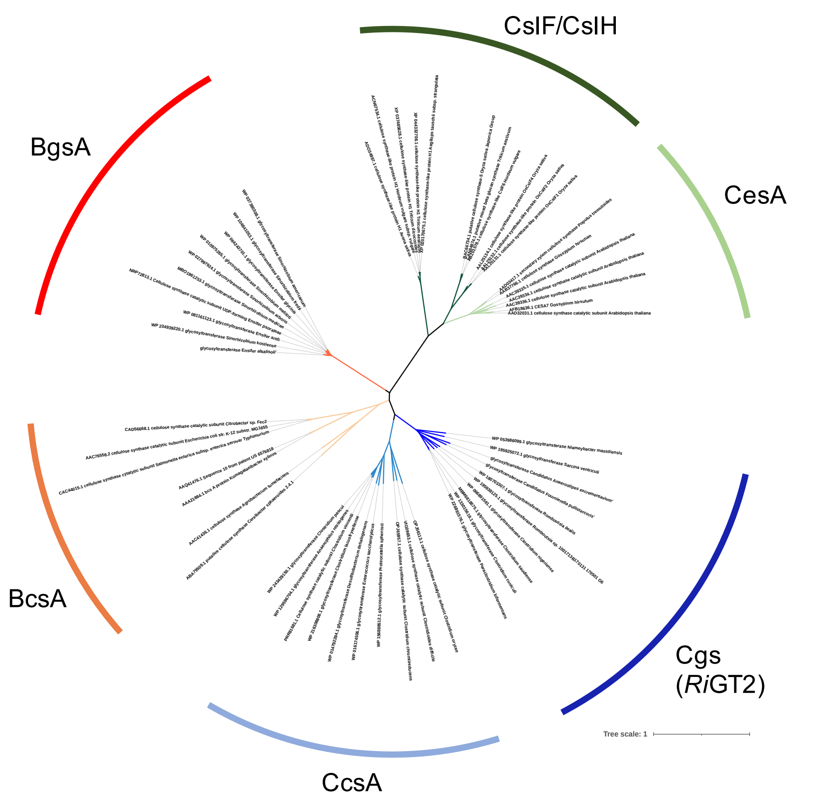
CslF/CslH
CesA
BgsA
BcsA
Cgs
(*Ri*GT2)
CcsA
Tree scale: 1

b.

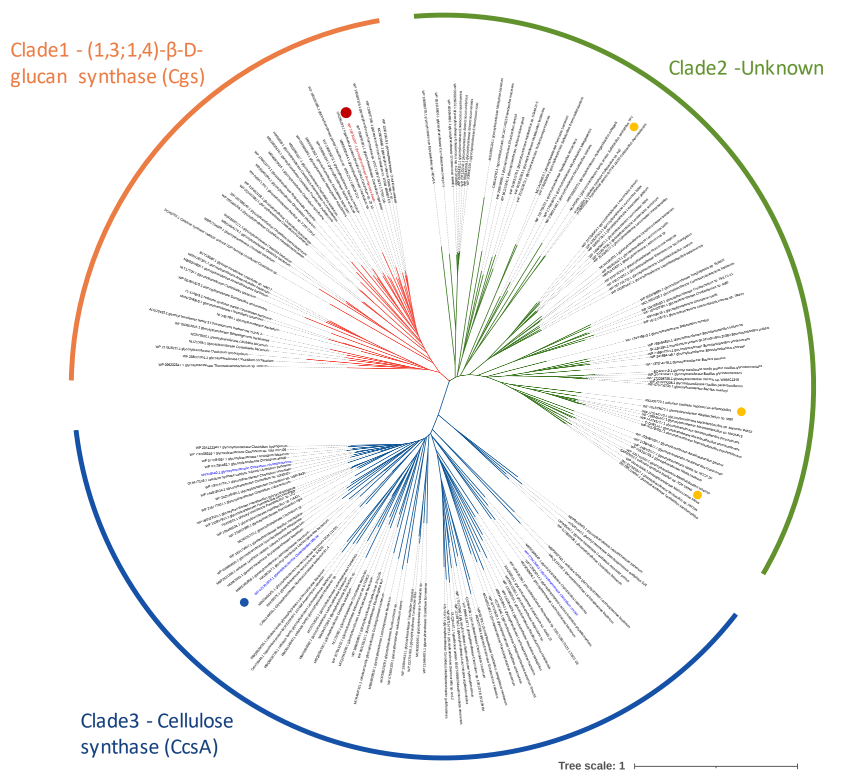
Clade1 - (1,3;1,4)-β-D-glucan synthase (Cgs)
Clade2 -Unknown
Clade3 - Cellulose synthase (CcsA)
Tree scale: 1

Supplementary Figure 16. Phylogenetic analyses of (1,3;1,4)-β-D-glucan and cellulose synthases.

a. Phylogenetic analysis of (1,3;1,4)-β-D-glucan synthases and cellulose synthases of grasses including cereals, and Gram-positive and Gram-negative bacteria. An unrooted neighbour-joining likelihood phylogeny of Gram-positive bacterial cellulose synthase (CcsA) and (1,3;1,4)-β-D-glucan synthases (Cgs); Gram-negative bacterial cellulose synthases (BcsA) and (1,3;1,4)-β-D-glucans synthase (Bgs); and grass cellulose synthases (CesA) and (1,3;1,4)-β-D-glucan synthases (CslF/ CslH) obtained using MEGA version X and iTOL. The scale bar shows the branch length. All protein entries are shown in **Supplementary Table 5.**

b. Phylogenetic analysis of (1,3;1,4)-β-D-glucan synthase (Cgs) and cellulose synthase (CcsA) in Gram-positive bacteria. An unrooted neighbour-joining likelihood phylogeny of Gram-positive bacterial (1,3;1,4)-β-D-glucan synthases (Cgs) and cellulose synthases (CcsA) obtained using MEGA version X and iTOL. *Ri*GT2 and *C. difficile* CcsA were used as the reference sequences and are marked with red and blue dots, respectively. Clade 1 (orange) contains *Ri*GT2 (1,3;1,4)-β-D-glucan synthase (red); clade 2 (green) contains GT2s with unknown functions, but with three proteins annotated as cellulose synthases (yellow dots); clade 3 (blue) contains three published putative cellulose synthases (CcsA) shown in blue text. The scale bar shows the branch length. All protein entries are shown in **Supplementary Table 6.**

a.

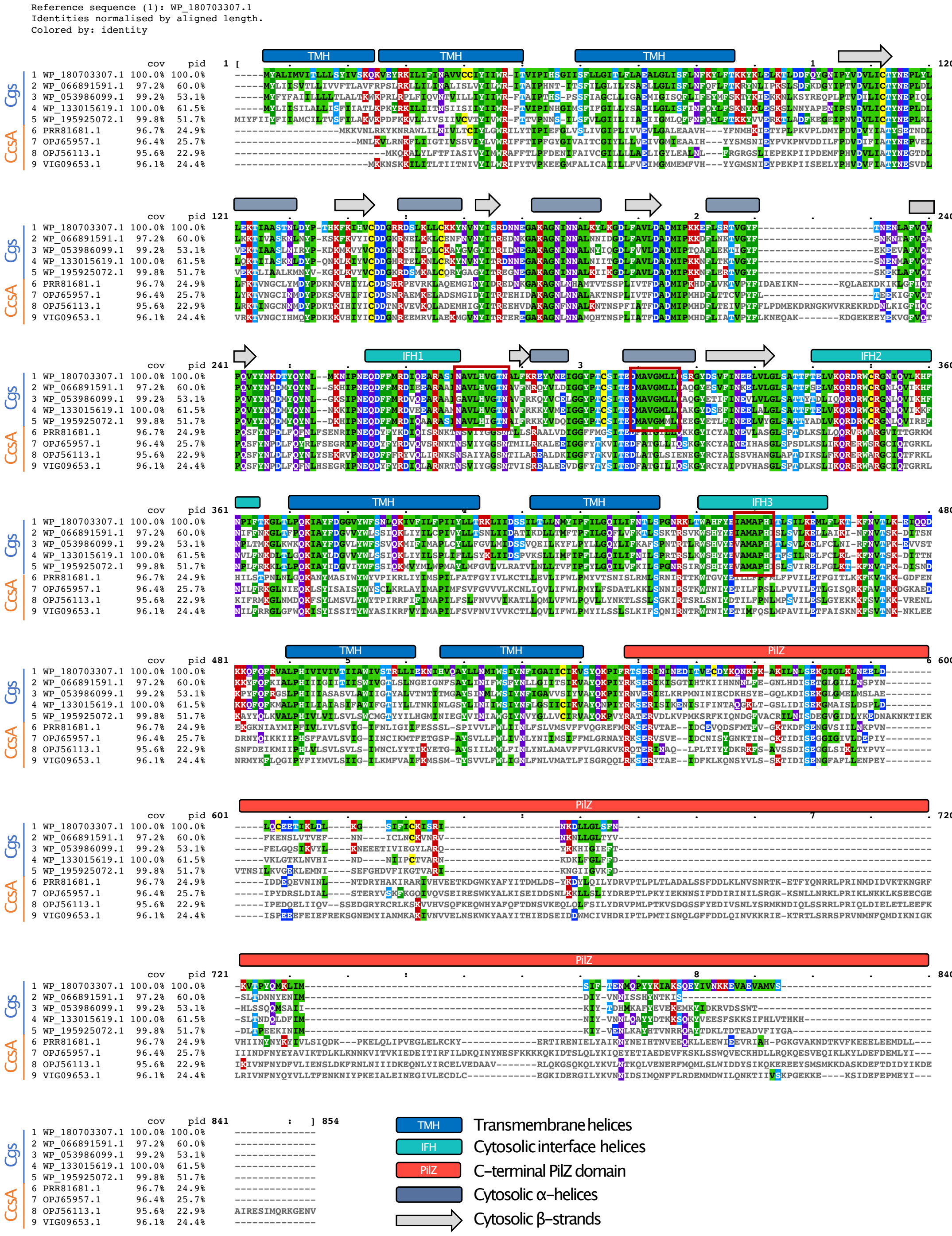
Reference sequence (1): WP_180703307.1
Identities normalised by aligned length.
Colored by: identity
Cgs
CcsA
1 WP_180703307.1 100.0% 100.0%
2 WP_066891591.1 97.2% 60.0%
3 WP_053986099.1 99.2% 53.1%
4 WP_133015619.1 100.0% 61.5%
5 WP_195925072.1 99.8% 51.7%
6 PRR81681.1 96.7% 24.9%
7 OPJ65957.1 96.4% 25.7%
8 OPJ56113.1 95.6% 22.9%
9 VIG09653.1 96.1% 24.4%
TMH
IFH1
IFH2
IFH3
PilZ
Transmembrane helices
Cytosolic interface helices
C-terminal PilZ domain
Cytosolic α-helices
Cytosolic β-strands

b.

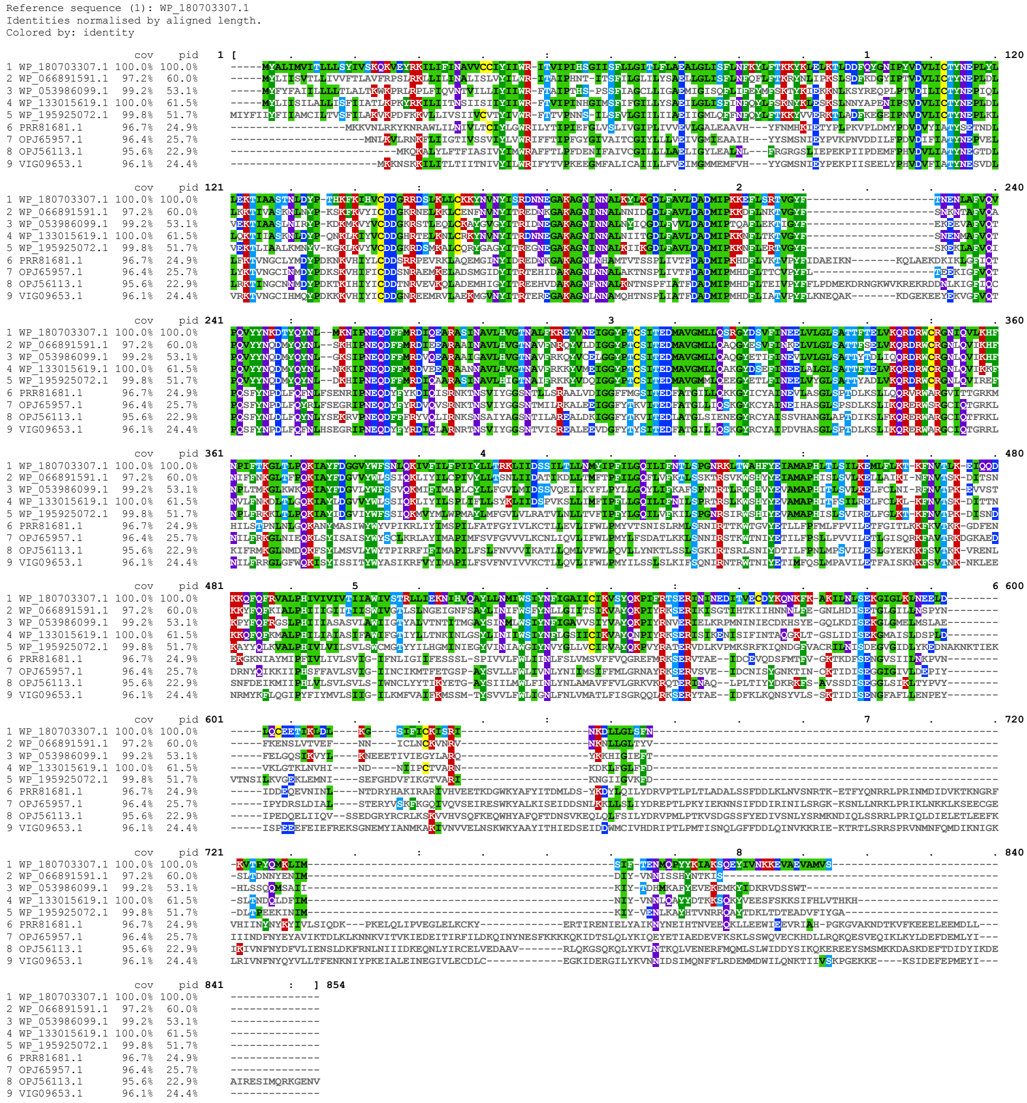

Supplementary Figure 17. Protein sequence alignments of Cgs and CcsA.

a. The sequence alignments of *Clostridia* β-glucan synthases (Cgs) and putative cellulose synthases of Gram-positive bacteria (CcsA). The alignment was prepared using Clustal. Proteins with the highest sequence identity to *Ri*GT2 (WP_180703307.1) from four species (*Clostridium nigeriense* [WP_066891591.1], *Niameybacter massiliensis* [WP_053986099.1], *Clostridium cuniculi* [WP_133015619.1] and *Sarcina ventriculi* [WP_195925072.1]), were selected as (1,3;1,4)-β-D-glucan synthase candidates. Four Clostridial cellulose synthases (CcsA) were also selected (*Clostridium vincentii* [PRR81681.1], *Clostridium chromiireducens* [OPJ65957.1], *Clostridium oryzae* [OPJ56113.1], and *Clostridioides difficile* [VIG09653.1]) (Scott *et al.* 2020)[45]. *Ri*GT2 (WP_180703307.1) was used as the reference sequence. The amino acid residues were marked if they were identical to the reference sequence. The secondary-structure elements shown at the top of the sequence were based on the *Cs*BcsA structure in **Supplementary Figure 10**.

Possible conserved regions are indicated with a red rectangle. All protein entries are shown in **Supplementary Table 6. b.** are the same sequence alignments but in text format.

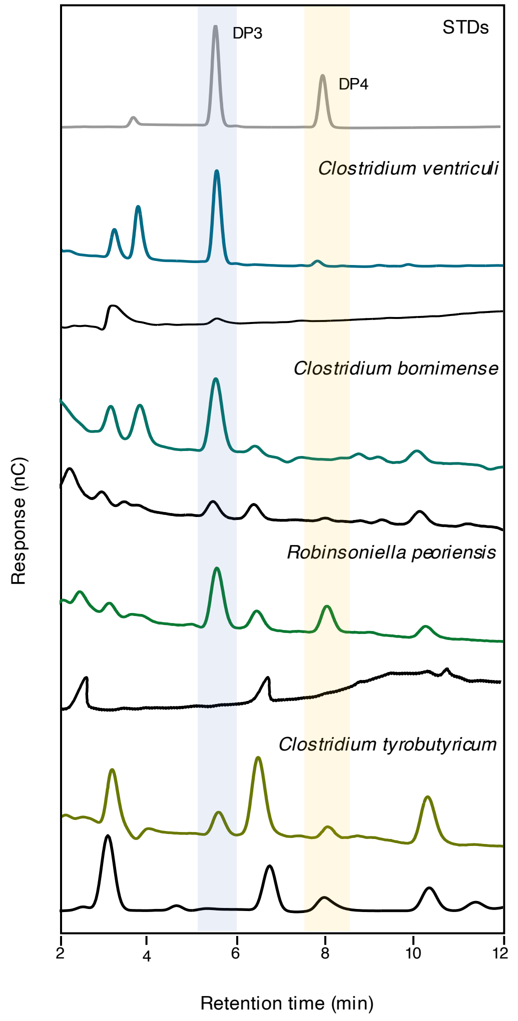

Supplementary Figure 18. (1,3;1,4)-β-D-glucan oligosaccharide profiles of four Gram-positive bacteria.

EPSs from *C. ventriculi, C. bornimence, R. peoriensis* and *C. tyrobutyrisum* were digested by lichenase and the hydrolysates were analysed by HPAEC-PAD and the retention times of peaks in the chromatograms compared with the pure standard (1,3;1,4)-β-D-glucan oligosaccharides DP3 and DP4 (STDs). Peaks corresponding to DP3 (marked in blue) were found in all four species, whereas peaks corresponding to DP4 (marked in yellow) were found in all species except *C. bornimence*. No lichenase controls are shown in black.

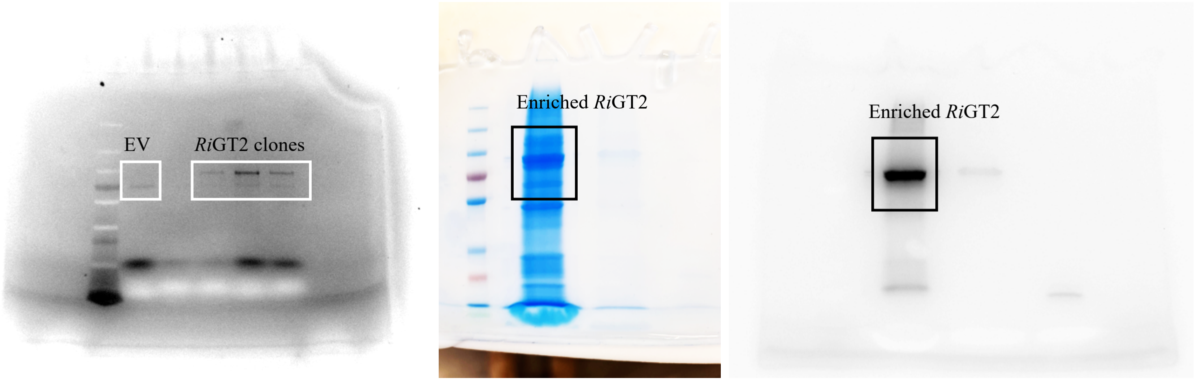

Supplementary Figure 19. Uncropped gels of **Supplementary Figure 3**.

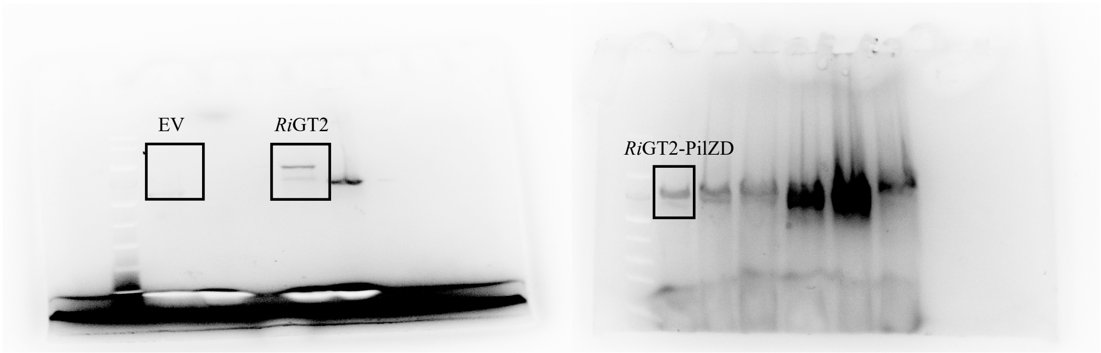

Supplementary Figure 20. Uncropped gels of **Supplementary Figure 15b**.

Supplementary Table 1. *GT2* genes present in the genome of *Romboutsia ilealis* annotated in the CAZy database.

Gene	Protein family	Protein accession
CRIB_1491	GT2	CED94098.1
CRIB_1504	GT2	CED94111.1
CRIB_1900	GT2	CED94506.1
CRIB_2065	GT2	CED94669.1
CRIB_856	GT2	CED93608.1

Supplementary Table 2. Expression levels of three *Ri*GT2 clones.

	Induced (RFU)	Non-induced (RFU)	Total protein expressed (mg)/litre of culture medium
*Ri*GT2 clone 1	22229	2336	1.21
*Ri*GT2 clone 2	27362	1982	1.63
*Ri*GT2 clone 3	17659	2375	0.93

*RFU, relative fluorescence units. Concentrations were calculated using whole cell fluorescence[31].

Supplementary Table 3. Linkage composition of the DP3 oligosaccharide obtained from lichenase digestion of the AIR of *Ri*GT2 LoGSA cells.

Linkages	3-Glucitol	t-Glc*p*	4-Glc*p*
**Mol%**	30.9 ± 1.1	34.5 ± 0.1	34.6 ± 1.1

*The reducing end of the oligosaccharide was initially reduced using $NaBD_4$

The linkage composition is given as mol%. The mol percentages are averages (± standard deviation) obtained from two separate experiments conducted on each sample.

Supplementary Table 4. Glucosyl-binding sites in *Cs*BcsA and in the predicted *Ri*GT2 model.

Glucosyl unit in cellulose (PDB 4HG6)	Side chains *Cs*BcsA, 4HG6 (secondary structure)	Side chains *Ri*GT2, model	Side chains *Hv*CslF6, 4DQK
Glc-18 (transfer site)	**Asp246 (β4)**<br>**His249 (loop β4/α4)**<br>**Gly319 (loop IFH1/β6)**<br>Ala344 (α5)<br>**Asp343 (α5)**	**Asp186**<br>Met189<br>**Gly256**<br>Asp280<br>Glu279	**Asp431**<br>**His434**<br>**Gly500**<br>Val635<br>**Asp634**
Glc-17	His276 (β5)<br>Tyr302 (IFH1)<br>**Trp383 (IFH2)**<br>Met387 (IFH2)	**Gln215**<br>Met239<br>**Trp320**<br>Asn324	**Gln464**<br>Phe483<br>**Trp676**<br>Ser680
Glc-16	**Phe301 (IFH1)**<br>**Trp417 (TMH5)**	**Phe288**<br>**Trp354**	**Phe482**<br>Pro712
Glc-15	Asn118 (TMH4)<br>**Ile305 (IFH1)**<br>Phe416 (TMH5)	Phe75<br>**Ile242**<br>**Tyr353**	Asp155<br>Thr486<br>**Tyr711**
Glc-14	Ile114 (TMH4)<br>Gln463 (TMH6)<br>**Glu480 (IFH3)**	**Ser71**<br>Phe400<br>**Glu417**	**Ser151**<br>Ala758<br>Trp779
Glc-13	Arg423 (TMH5)<br>Ser459 (TMH6)<br>Glu477 (IFH3)	Lys360<br>Gln396<br>His414	Leu718<br>Leu754<br>Gln777
Glc-12	**Phe426 (TMH5)**<br>Tyr455 (TMH6)	**Phe363**<br>Phe392	Tyr721<br>Val750
Glc-11	Met452 (TMH6)<br>**Trp558 (TMH8)**	Tyr389<br>**Trp495**	Leu747<br>Phe872
Glc-10	**Leu104 (TMH4)**<br>**Pro430 (TMH5)**<br>Phe441 (loop TMH5/YMH6)<br>Val554 (TMH8)<br>Val555 (TMH8)	**Leu61**<br>**Pro367**<br>Ile378<br>Leu491<br>Asn492	Ser141<br>**Pro725**<br>Val736<br>Gly868<br>Gly869
Glc-9	**Tyr433 (TMH5)**<br>**Val551 (TMH8)**	**Tyr370**<br>Ala488	Ser728<br>**Val866**
Glc-8	Glu439 (loop TMH5/YMH6)<br>Asp548 (TMH8)	Leu376<br>His485	Phe734<br>–

Conserved residue pairs between at least two proteins are in bold face: all proteins, blue; *Cs*BcsA-*Ri*GT2, green; *Cs*BcsA-*Hv*CslF6, orange; *Ri*GT2-*Hv*CslF6, purple.

Supplementary Supplementary Table 5. Protein entries used for the phylogenetic analysis in Supplementary Figure 16a.

Accession number	Annotation	Species	Database	Comments
		**CcsA**		
PRR81681.1	Cellulose synthase catalytic subunit	*Clostridium vincentii*	CAZy	
OPJ65957.1	Cellulose synthase catalytic subunit	*Clostridium chromiireducens*	CAZy	
OPJ56113.1	Cellulose synthase catalytic subunit	*Clostridium oryzae*	CAZy	
VIG09653.1	Cellulose synthase catalytic subunit	*Clostridioides difficile*	CAZy	
WP_243429150.1	glycosyltransferase	*Clostridium pascui*	NCBI	99% coverage and 79% identity against query sequence PRR81681.1
WP_216308808	glycosyltransferase	*Clostridium lacusfryxellense*	NCBI	100% coverage and 79% identity against query sequence PRR81681.1
WP_129596704.1	glycosyltransferase	*Anaerophilus nitritogenes*	NCBI	100% coverage and 78% identity against query sequence PRR81681.1
WP_014792294.1	glycosyltransferase	*Desulfitobacterium dehalogenans*	NCBI	95% coverage and 64% identity against query sequence PRR81681.1
WP_156888512.1	glycosyltransferase	*Proteocatella sphenisci*	NCBI	97% coverage and 59% identity against query sequence PRR81681.1
WP_016174506.1	glycosyltransferase	*Enterococcus saccharolyticus*	NCBI	96% coverage and 57% identity against query sequence PRR81681.1
		**Cgs**		
WP_180703307.1	Cellulose synthase catalytic subunit [UDP-forming]	*Romboutsia ilealis*	CAZy	
WP_133015619.1	glycosyltransferase	*Clostridium cuniculi*	NCBI	97% coverage and 63% identity against query sequence WP_180703307.1
WP_195938125.1	glycosyltransferase	*Romboutsia sp. 1001713B170131_170501_G6*	NCBI	99% coverage and 63% identity against query sequence WP_180703307.1
WP_066891591.1	glycosyltransferase	*Clostridium nigeriense*	NCBI	85% coverage and 60% identity against query sequence WP_180703307.1
WP_226891576.1	glycosyltransferase	*Paraclostridium bifermentans*	NCBI	89% coverage and 64% identity against query sequence WP_180703307.1
MBU3805678.1	glycosyltransferase	*Candidatus Fournierella pullistercoris*	NCBI	97% coverage and 58% identity against query sequence WP_180703307.1
WP_195925072.1	glycosyltransferase	*Sarcina ventriculi*	NCBI	96% coverage and 54% identity against query sequence WP_180703307.1
MBM6818575.1	glycosyltransferase	*Clostridium saudiense*	NCBI	82% coverage and 64% identity against query sequence WP_180703307.1

HIX66809.1	MAG TPA: glycosyltransferase	*Candidatus Anaerostipes excrementavium*	NCBI	96% coverage and 54% identity against query sequence WP_180703307.1
WP_053986099.1	glycosyltransferase	*Niameybacter massiliensis*	NCBI	95% coverage and 54% identity against query sequence WP_180703307.1
**BcsA**				
AAC41436.1	cellulose synthase	*Agrobacterium tumefaciens*	CAZy	
ABA79509.1	putative cellulose synthase	*Cereibacter sphaeroides 2.4.1*	CAZy	
CAD56668.1	cellulose synthase catalytic subunit	*Citrobacter sp. Fec2*	CAZy	
AAC76558.2	cellulose synthase catalytic subunit	*Escherichia coli str. K-12 substr. MG1655*	CAZy	
AAA21884.1	bcs A protein	*Komagataeibacter xylinus*	CAZy	
CAC44015.1	cellulose synthase catalytic subunit	*Salmonella enterica subsp. enterica serovar Typhimurium*	CAZy	
**BgsA**				
WP_010975265.1	glycosyltransferase	*Sinorhizobium meliloti*	NCBI	
WP_027997924.1	glycosyltransferase	*Sinorhizobium arboris*	NCBI	100% coverage and 97% identity against query sequence WP_010975265.1
MBO1961253.1	glycosyltransferase	*Sinorhizobium medicae*	NCBI	100% coverage and 95% identity against query sequence WP_010975265.1
WP_037380308.1	glycosyltransferase	*Sinorhizobium americanum*	NCBI	100% coverage and 88% identity against query sequence WP_010975265.1
WP_104841053.1	glycosyltransferase	*Sinorhizobium fredii*	NCBI	100% coverage and 88% identity against query sequence WP_010975265.1
WP_234939220.1	glycosyltransferase	*Sinorhizobium kostiense*	NCBI	100% coverage and 84% identity against query sequence WP_010975265.1
WP_081161123.1	glycosyltransferase	*Ensifer aridi*	NCBI	100% coverage and 88% identity against query sequence WP_010975265.1
NRP72813.1	Cellulose synthase catalytic subunit [UDP-forming]	*Ensifer psoraleae*	NCBI	99% coverage and 89% identity against query sequence WP_010975265.1
WP_064243701.1	glycosyltransferase	*Ensifer glycinis*	NCBI	100% coverage and 87% identity against query sequence WP_010975265.1
MCG5480378.1	glycosyltransferase	*Ensifer alkalisoli*	NCBI	100% coverage and 86% identity against query sequence WP_010975265.1
**CesA**				
AAC39336.1	cellulose synthase catalytic subunit	*Arabidopsis thaliana*	CAZy	
AAD32031.1	cellulose synthase catalytic subunit	*Arabidopsis thaliana*	CAZy	

AAC39335.1	cellulose synthase catalytic subunit	*Arabidopsis thaliana*	CAZy	
AAB37766.1	cellulose synthase catalytic subunit	*Gossypium hirsutum*	CAZy	
AFB18636.1	CESA7	*Gossypium hirsutum*	CAZy	
AAD03417.1	CesA secondary xylem cellulose synthase	*Populus tremuloides*	CAZy	
**CslF**				
ABZ01578.1	cellulose synthase-like CslF6	*Hordeum vulgare*	CAZy	
CAN84874.1	CslF6 putative (1,3;1,4)-β-D-glucan synthase	*Triticum aestivum*	CAZy	
AAL25134.1	cellulose synthase-like protein OsCslF4	*Oryza sativa*	CAZy	
AAL25131.1	cellulose synthase-like protein OsCslF1	*Oryza sativa*	CAZy	
AAL25132.1	cellulose synthase-like protein OsCslF2	*Oryza sativa*	CAZy	
BAC66734.1	CslF6 putative cellulose synthase-5	*Oryza sativa Japonica Group*	CAZy	
**CslH**				
ACN67534.1	cellulose synthase-like protein H1	*Hordeum vulgare subsp. vulgare*	CAZy	
XP_020170675.1	cellulose synthase-like protein H2	*Aegilops tauschii subsp. strangulata*	NCBI	100% coverage and 93% identity against query sequence ACN67534.1
XP_037485628.1	cellulose synthase-like protein H3	*Triticum dicoccoides*	NCBI	100% coverage and 93% identity against query sequence ACN67534.1
XP_044332708.1	cellulose synthase-like protein H4	*Triticum aestivum*	NCBI	100% coverage and 93% identity against query sequence ACN67534.1
ADO34997.1	cellulose synthase-like protein H5	*Avena sativa*	NCBI	100% coverage and 83% identity against query sequence ACN67534.1

Supplementary Table 6. Protein entries for the phylogenetic analysis in Supplementary Figure 16b.

Assession	Annotation	Species
**Clade 1**		
WP_096232347.1	glycosyltransferase	Thermoanaerobacterium sp. RBIITD
WP_238021861.1	glycosyltransferase	*Clostridium cochlearium*
WP_217818211.1	glycosyltransferase	*Clostridium tyrobutyricum*
NLI21588.1	glycosyltransferase	*Clostridiales bacterium*
NCB73502.1	glycosyltransferase	*Clostridia bacterium*
WP_083803616.1	glycosyltransferase	*Ethanoligenens harbinense*
ADU26422.1	glycosyl transferase family 2	*Ethanoligenens harbinense* YUAN-3
NCA92788.1	glycosyltransferase	bacterium
MBN2258955.1	glycosyltransferase	Clostridiales bacterium
PLX34863.1	cellulose synthase	partial Clostridiales bacterium
WP_063965525.1	glycosyltransferase	*Domibacillus aminovorans*
NLT17709.1	glycosyltransferase	Clostridiales bacterium
MBI9050800.1	glycosyltransferase	Anaerolineaceae bacterium
MBN1267381.1	glycosyltransferase	Anaerolineales bacterium
BCY18588.1	glycosyltransferase	*Leptolinea* sp. HRD-7
SCI49763.1	Cellulose synthase catalytic subunit UDP-forming	uncultured *Clostridium* sp.
MBR2704500.1	glycosyltransferase	Clostridia bacterium
MBR1654174.1	glycosyltransferase	Clostridia bacterium
MBR2240221.1	glycosyltransferase	Clostridia bacterium
WP_055069358.1	glycosyltransferase	*Clostridium massiliamazoniense*
WP_002598145.1	glycosyltransferase	*Clostridium thermobutyricum*
WP_051483683.1	glycosyltransferase	*Clostridium bornimense*
WP_216463108.1	glycosyltransferase	*Clostridium bornimense*
WP_049671702.1	glycosyltransferase	*Bacillus* sp. FJAT-27916
MBS5082054.1	glycosyltransferase	Clostridiales bacterium
WP_138001473.1	glycosyltransferase	*Robinsoniella peoriensis*
MBM6929516.1	glycosyltransferase	*Clostridium spiroforme*
MBU3805678.1	glycosyltransferase	*Candidatus fournierella pullistercoris*
HIX66809.1	glycosyltransferase	*Candidatus anaerostipes excrementavium*
MBS5389117.1	glycosyltransferase	*Clostridiales bacterium*
WP_053986099.1	glycosyltransferase	*Niameybacter massiliensis*
MBS5799352.1	glycosyltransferase	Clostridiales bacterium
WP_058991834.1	glycosyltransferase	*Sarcina ventriculi*
WP_195925072.1	glycosyltransferase	*Sarcina ventriculi*

WP_195931698.1	glycosyltransferase	partial *Clostridium* sp. 1001270J 160509 D11
MBE6050444.1	glycosyltransferase	*Clostridium* sp.
OKZ86193.1	hypothetical protein BHW04 07245	*Clostridium* sp. 29 15
WP_180703307.1	glycosyltransferase	*Romboutsia ilealis*
WP_066891591.1	glycosyltransferase	*Clostridium nigeriense*
WP_195938125.1	glycosyltransferase	*Romboutsia* sp. 1001713B170131 170501 G6
WP_195987356.1	glycosyltransferase	*Clostridium* sp. D53t1 180928 C8
MCI9069666.1	glycosyltransferase	*Clostridium* sp.
WP_133015619.1	glycosyltransferase	*Clostridium cuniculi*
**Clade 2**		
WP_166081879.1	glycosyltransferase	*Erysipelothrix* sp. HDW6A
WP_051915606.1	glycosyltransferase	*Carnobacterium divergens*
WP_069664692.1	glycosyltransferase	*Enterococcus termitis*
WP_086329102.1	glycosyltransferase	*Enterococcus* sp. 4G2 DIV0659
WP_069634311.1	glycosyltransferase	*Enterococcus quebecensis*
WP_207120243.1	glycosyltransferase	*Enterococcus ureilyticus*
WP_086346359.1	glycosyltransferase	*Enterococcus termitis*
WP_208930334.1	glycosyltransferase	*Enterococcus rotai*
MBI3605186.1	glycosyltransferase	*Nitrospirae bacterium*
OMG48792.1	hypothetical protein BK140 14525	*Paenibacillus macerans*
WP_200760060.1	glycosyltransferase	*Effusibacillus dendaii*
WP_161263240.1	glycosyltransferase	*Heliomicrobium gestii*
WP_245031375.1	glycosyltransferase	*Halobacillus* sp. SSHM10-5
WP_066228353.1	glycosyltransferase	*Metabacillus fastidiosus*
WP_251522019.1	glycosyltransferase	*Robertmurraya korlensis*
MCL4494059.1	glycosyltransferase	*Firmicutes bacterium*
WP_076005804.1	glycosyltransferase	*Sulfobacillus thermosulfidooxidans*
WP_132768261.1	glycosyltransferase	*Tepidibacillus fermentans*
WP_217994673.1	glycosyltransferase	*Alicyclobacillus kakegawensis*
WP_236814162.1	glycosyltransferase	*Alicyclobacillus tolerans*
MBT9282300.1	glycosyltransferase	*Hydrogenibacillus schlegelii*
AEJ40305.1	glycosyl transferase family protein	*Sulfobacillus acidophilus* TPY
POB11864.1	cellulose synthase	*Sulfobacillus* sp. hq2
AUW95299.1	hypothetical protein BXT84 16150	*Sulfobacillus thermotolerans*
WP_216769954.1	glycosyltransferase	*Leuconostoc citreum*
WP_010007313.1	glycosyltransferase	*Leuconostoc fallax*
WP_089997761.1	glycosyltransferase	*Leuconostoc gelidum*
WP_148606451.1	glycosyltransferase	*Leuconostoc litchii*
WP_211637090.1	glycosyltransferase	*Leuconostoc suionicum*

WP_252169177.1	glycosyltransferase	*Leuconostoc mesenteroides*
MCH4169501.1	glycosyltransferase	*Streptococcaceae bacterium*
WP_085624023.1	glycosyltransferase	*Lactococcus lactis*
MBR2541937.1	glycosyltransferase	*Lactococcus* sp.
WP_070792914.1	glycosyltransferase	*Floricoccus tropicus*
WP_016174251.1	glycosyltransferase	*Enterococcus saccharolyticus*
WP_057738792.1	glycosyltransferase	*Liquorilactobacillus uvarum*
WP_261909447.1	glycosyltransferase	*Liquorilactobacillus satsumensis*
WP_055859999.1	glycosyltransferase	*Yonghaparkia* sp. Soil809
MCL5052835.1	glycosyltransferase	Gammaproteobacteria bacterium
WP_134508283.1	glycosyltransferase	*Cryobacterium* sp. RHLT2-21
WP_104100989.1	glycosyltransferase	*Cryobacterium* sp. M96
MPV89615.1	glycosyltransferase	*Georgenia ruanii*
WP_167139576.1	glycosyltransferase	*Diaminobutyricimonas* sp. TR449
WP_174496823.1	glycosyltransferase	*Salirhabdus euzebyi*
WP_253054818.1	glycosyltransferase	*Sporolactobacillus kofuensis*
GGL56196.1	hypothetical protein GCM10007968 20360	*Sporolactobacillus putidus*
WP_239984769.1	glycosyltransferase	*Sporolactobacillus pectinivorans*
WP_241654749.1	glycosyltransferase	*Sporolactobacillus shoreae*
WP_137054248.1	glycosyltransferase	*Bacillus pumilus*
SCA88359.1	glycosyl transferase family protein	*Bacillus glycinifermentans*
WP_247069843.1	glycosyltransferase	*Bacillus glycinifermentans*
WP_172298738.1	glycosyltransferase	*Bacillus* sp. WMMC1349
WP_076759736.1	glycosyltransferase	*Bacillus swezeyi*
WP_216919206.1	glycosyltransferase	*Bacillus paralicheniformis*
RSU08770.1	cellulose synthase	*Vagococcus entomophilus*
WP_161879625.1	glycosyltransferase	*Alkalibacterium* sp. MB6
WP_225744710.1	glycosyltransferase	*Marinilactibacillus* sp. Marseille-P9653
WP_208560442.1	glycosyltransferase	*Marinilactibacillus* sp. M4U5P12
WP_143745272.1	glycosyltransferase	*Marinilactibacillus piezotolerans*
TLQ08134.1	glycosyltransferase	*Marinilactibacillus psychrotolerans*
WP_091760002.1	glycosyltransferase	*Marinilactibacillus psychrotolerans*
WP_203088826.1	glycosyltransferase	*Alkalihalobacillus gibsonii*
WP_113869823.1	glycosyltransferase	*Paraliobacillus ryukyuensis*
WP_248562737.1	glycosyltransferase	*Niallia* sp. NCCP-28
WP_016202705.1	glycosyltransferase	*Niallia nealsonii*
WP_241741820.1	glycosyltransferase	*Alkalihalobacillus lehensis*
GAF20063.1	cellulose synthase	*Bacillus* sp. JCM 19046
WP_091615096.1	glycosyltransferase	*Marinococcus luteus*

WP_217321847.1	glycosyltransferase	*Terribacillus* sp. DMT04
WP_095218650.1	glycosyltransferase	*Terribacillus saccharophilus*
**Clade 3**		
MBP9630560.1	glycosyltransferase	Leptotrichiaceae bacterium
AFM41440.1	glycosyltransferase	*Desulfosporosinus acidiphilus* SJ4
OFW35083.1	glycosyltransferase	*Candidatus Aquicultor primus*
WP_077895008.1	glycosyltransferase	*Clostridium felsineum*
MBP9997461.1	cellulase family glycosylhydrolase	Lachnospiraceae bacterium
MBQ1194266.1	glycosyltransferase	Lachnospiraceae bacterium
MBS5599849.1	glycosyltransferase	*Coprobacillus cateniformis*
WP_079428453.1	glycosyltransferase	*Clostridium oryzae*
WP_029505014.1	glycosyltransferase	*Lachnoclostridium phytofermentans*
MCI9052604.1	glycosyltransferase	Lachnospiraceae bacterium
WP_195939288.1	glycosyltransferase	*Romboutsia* sp. 1001713B170131 170501 G6
RHO58815.1	glycosyltransferase	*Eubacterium* sp. AM05-23
MCR5101651.1	glycosyltransferase	*Butyrivibrio* sp.
WP_130862559.1	glycosyltransferase	*Bacilliculturomica massiliensis*
WP_262064839.1	glycosyltransferase	*Aequitasia blattaphilus*
MBO4309940.1	glycosyltransferase	Lachnospiraceae bacterium
UQZ87848.1	glycosyltransferase	Deltaproteobacteria bacterium Smac51
WP_015710843.1	glycosyltransferase	*Leadbettera azotonutricia*
MCD8351987.1	glycosyltransferase	Planctomycetaceae bacterium
HJA90788.1	glycosyltransferase	*Candidatus Jeotgalibaca merdavium*
RGW05786.1	glycosyltransferase	*Streptococcus salivarius*
QOX64858.1	glycosyltransferase	Clostridiales bacterium
WP_196001637.1	glycosyltransferase	*Clostridium* sp. 1001271B 151109 B4
WP_179237502.1	glycosyltransferase	*Sedimentibacter hydroxybenzoicus*
WP_120197652.1	glycosyltransferase	*Lacrimispora algidixylanolytica*
OUQ34487.1	hypothetical protein B5E75 06680	*Massilimicrobiota timonensis*
WP_087167871.1	glycosyltransferase	*Drancourtella* sp. An12
HIV41172.1	glycosyltransferase	Candidatus Mediterraneibacter guildfordensis
WP_216464074.1	glycosyltransferase	*Clostridium bornimense*
MCI9350695.1	glycosyltransferase	*Turicibacter* sp.
WP_212724769.1	glycosyltransferase	*Turicibacter bilis*
WP_195944431.1	glycosyltransferase	*Turicibacter sanguinis*
WP_075818130.1	glycosyltransferase	*Ileibacterium valens*
MCR5652929.1	glycosyltransferase	*Ruminococcus* sp.
MBD8939334.1	glycosyltransferase	Lachnospiraceae bacterium
MCR4647121.1	cellulase family glycosylhydrolase	Oscillospiraceae bacterium

WP_069151110.1	glycosyltransferase	*Eisenbergiella tayi*
WP_186865999.1	glycosyltransferase	*Roseburia* sp. BX1005
MCQ2549293.1	glycosyltransferase	Lachnospiraceae bacterium
WP_207941222.1	glycosyltransferase	*Enterococcus* sp. DIV2402
NLL79092.1	glycosyltransferase	Clostridiales bacterium
MBQ8094330.1	glycosyltransferase	Clostridia bacterium
MBO4457288.1	glycosyltransferase	*Butyrivibrio* sp.
MBP5385562.1	glycosyltransferase	Lachnospiraceae bacterium
MCI6713543.1	glycosyltransferase	Lachnospiraceae bacterium
MBO6136540.1	cellulase family glycosylhydrolase	*Fibrobacter* sp.
MBQ6635738.1	cellulase family glycosylhydrolase	Lachnospiraceae bacterium
ORX78046.1	hypothetical protein BCR32DRAFT 247468	*Anaeromyces robustus*
MBQ9828035.1	cellulase family glycosylhydrolase	Lachnospiraceae bacterium
CAB1246630.1	glycosyltransferase	Ruminococcaceae bacterium BL-4
RPA59575.1	glycosyltransferase	*Aerococcus* sp. SJQ22
MBG9984301.1	glycosyltransferase	Aerococcaceae bacterium DSM 111022
WP_021391996.1	glycosyltransferase	Clostridioides difficile
HAU86397.1	glycosyltransferase	Lachnospiraceae bacterium
MBE5959959.1	glycosyltransferase	Lachnospiraceae bacterium
HAM63333.1	glycosyltransferase	Erysipelotrichaceae bacterium
MBP2631288.1	cellulose synthase catalytic subunit	Firmicutes bacterium
WP_093669839.1	glycosyltransferase	*Sporolactobacillus nakayamae*
WP_163179807.1	glycosyltransferase	*Bacillus mesophilus*
MCI6275716.1	glycosyltransferase	*Clostridium* sp.
WP_218827896.1	glycosyltransferase	*Paenibacillus rigui*
WP_108466534.1	glycosyltransferase	*Paenibacillus* sp. CAA11
PIH56018.1	glycosyltransferase	*Paenibacillus* sp. LK1
WP_110897933.1	glycosyltransferase	*Paenibacillus barcinonensis*
WP_090923110.1	glycosyltransferase	*Paenibacillus polysaccharolyticus*
WP_235777807.1	glycosyltransferase	*Clostridium culturomicium*
WP_242946059.1	glycosyltransferase	*Clostridium* sp. DSM 8431
WP_244833814.1	glycosyltransferase	*Clostridium* sp. BJN0001
WP_230142705.1	glycosyltransferase	*Clostridium neonatale*
OOM77166.1	cellulose synthase catalytic subunit	*Clostridium puniceum*
MVX65640.1	glycosyltransferase	*Clostridium chromiireducens*
WP_035796462.1	glycosyltransferase	*Clostridium akagii*
WP_077894097.1	glycosyltransferase	*Clostridium felsineum*
WP_234122349.1	glycosyltransferase	*Clostridium hydrogenum*
WP_238906319.1	glycosyltransferase	*Clostridium* sp. YIM B02506

Supplementary Table 7. Primers used for the *RiGT2* and *RiPilZ* transformation.

Primer names	Sequences
Ri_pDDGFP2_F	ACCCCGGATTCTAGAACTAGTGGATCCCCCATGTACGCCTTGATTATGGTCATC
Ri_pDDGFP2_R	AAATTGACCTTGAAAATATAAATTTTCCCCAGACACCATGGCGACTTCAGC
RiPilZ_pDDGFP2_F	ACCCCGGATTCTAGAACTAGTGGATCCCCCATGCCTATTTTCAGGACCTCCGA
