# Supplementary material for: The Gram-positive bacterium Romboutsia ilealis harbors a polysaccharide synthase that can produce (1,3;1,4)-β-d-glucans
Source: Nat Commun. 2023 Jul 27;14:4526. doi: 10.1038/s41467-023-40214-z (PMC10374906; doi:10.1038/s41467-023-40214-z)
Supplement: Supplementary file 3 — Reporting Summary [file 41467_2023_40214_MOESM3_ESM.pdf]

nature portfolio

Corresponding author(s): Yves S. Y. Hsieh

Last updated by author(s): May 26, 2023

Reporting Summary

Nature Portfolio wishes to improve the reproducibility of the work that we publish. This form provides structure for consistency and transparency in reporting. For further information on Nature Portfolio policies, see our Editorial Policies and the Editorial Policy Checklist.

Please do not complete any field with "not applicable" or n/a. Refer to the help text for what text to use if an item is not relevant to your study.
For final submission: please carefully check your responses for accuracy; you will not be able to make changes later.

Statistics

For all statistical analyses, confirm that the following items are present in the figure legend, table legend, main text, or Methods section.

n/a	Confirmed	
☐	☒	The exact sample size (*n*) for each experimental group/condition, given as a discrete number and unit of measurement
☐	☒	A statement on whether measurements were taken from distinct samples or whether the same sample was measured repeatedly The statistical test(s) used AND whether they are one- or two-sided<br>*Only common tests should be described solely by name; describe more complex techniques in the Methods section.*
☒	☐	A description of all covariates tested
☒	☐	A description of any assumptions or corrections, such as tests of normality and adjustment for multiple comparisons
	☒	A full description of the statistical parameters including central tendency (e.g. means) or other basic estimates (e.g. regression coefficient) AND variation (e.g. standard deviation) or associated estimates of uncertainty (e.g. confidence intervals)
☒	☐	For null hypothesis testing, the test statistic (e.g. *F*, *t*, *r*) with confidence intervals, effect sizes, degrees of freedom and *P* value noted<br>*Give P values as exact values whenever suitable.*
☒	☐	For Bayesian analysis, information on the choice of priors and Markov chain Monte Carlo settings
☒	☐	For hierarchical and complex designs, identification of the appropriate level for tests and full reporting of outcomes
☒	☐	Estimates of effect sizes (e.g. Cohen's *d*, Pearson's *r*), indicating how they were calculated
☒	☐	

Our web collection on statistics for biologists contains articles on many of the points above.

Software and code

Policy information about availability of computer code

Data collection	National Center For Biotechnology Information database (NCBI); Protein Data Bank (PDB); Carbohydrate-Active enZYmes (CAZy) Database
Data analysis	Bioinformatic tools used include EMBL-Web tool; MEGA X; iTol Online tool; ExPASy Compute pI/Mw, Chimera; Alphafold2 were used for data analysis. Confocal image acquisition was performed with a Leica TCS SP5 microscope using Leica microsystem LAS AF software.<br>Electron miscroscopy image acquisition was performed on an Orius SC200 CCD camera (Gatan Inc/Blue-Scientific), using Gatan Digital Micrograph software.<br>SPR data were plotted in a resonance unit against time sensorgram and analyzed using the Biaevaluation software.<br>ITC data were plotted by Microcal PEAQ-ITC analysis software.<br>Dionex HPAEC-PAD chromatogram was analyzed by Chromelion software version 7.<br>MALDI-TOF MS spectra was analyzed by Applied Biosystems 4000 Series Explorer software.<br>UCSF ChimeraX, PyMOL (structure visualisation = structure figures).<br>SWISS-MODEL, AlphaFol2 (through Colab) (generation of theoretical RiGT2 models).<br>Clustal Omega (pre-MSA for CsBcsA and RiGT2).<br>Coot 0.9 (manual structure-based adjustment of MSA for CsBcsA and RiGT2).<br>ESPript3.0 (MSA visualisation).<br>NCBI Sequence Viewer, https://www.ncbi.nlm.nih.gov/projects/sviewer/ (genome analysis)<br>ExPASy Compute pI/Mw, https://web.expasy.org/compute_pi/

Data

Policy information about availability of data

All manuscripts must include a data availability statement. This statement should provide the following information, where applicable:

- Accession codes, unique identifiers, or web links for publicly available datasets
- A description of any restrictions on data availability
- For clinical datasets or third party data, please ensure that the statement adheres to our policy

All the databases/datasets used in the study along with appropriately accessible links/accession-codes in the manuscript under the “Data availability”. The data that support the findings of this study are also available within the article and its Supplementary Information.

Human research participants

Policy information about studies involving human research participants and Sex and Gender in Research.

Reporting on sex and gender	N/A
Population characteristics	N/A
Recruitment	N/A
Ethics oversight	N/A

Note that full information on the approval of the study protocol must also be provided in the manuscript.

Field-specific reporting

Please select the one below that is the best fit for your research. If you are not sure, read the appropriate sections before making your selection.

☒ Life sciences ☐ Behavioural & social sciences ☐ Ecological, evolutionary & environmental sciences

For a reference copy of the document with all sections, see nature.com/documents/nr-reporting-summary-flat.pdf

Life sciences study design

All studies must disclose on these points even when the disclosure is negative.

Sample size	The experiment was performed in triplicates and the data were analyzed descriptively.
Data exclusions	No data exclusion.
Replication	All experimental measurements were performed in triplicate, except for sugar linkage analysis which was done in duplicate. Error bars represent SD. For the confocal and electron microscopies, at least three slides/grids were prepared independently. All attempts of replication were successful and gave similar results.
Randomization	Randomization was not deemed relevant due study design does not involve a comparison or intervention that requires random assignment.
Blinding	Blinding was not performed in this study due to the nature of the experimental design and the absence of subjective or biased outcome assessments. The study primarily relied on objective measurements and data collection methods, eliminating the potential for observer bias or subjective interpretation. As a result, blinding was not considered necessary to ensure the integrity of the study's results

Reporting for specific materials, systems and methods

We require information from authors about some types of materials, experimental systems and methods used in many studies. Here, indicate whether each material, system or method listed is relevant to your study. If you are not sure if a list item applies to your research, read the appropriate section before selecting a response.

Materials & experimental systems

n/a	Involved in the study
☐	☒ Antibodies
☒	☐ Eukaryotic cell lines
☒	☐ Palaeontology and archaeology
☒	☐ Animals and other organisms
☒	☐ Clinical data
☒	☐ Dual use research of concern
☒	☐

Methods

n/a	Involved in the study
☒	☐ ChIP-seq
☒	☐ Flow cytometry
☒	☐ MRI-based neuroimaging

Antibodies

Antibodies used

Monoclonal Mouse IgG Antibody to (1,3, 1,4)-β-glucan of hybridoma BG1 (Cat 400-3; BioSupplies Australia Pty Ltd).
Goat anti-mouse secondary antibody conjugated to 12 nm colloidal gold particles (Cat 115-205-146; Jackson ImmunoResearch, PA, USA).
Goat Anti-Mouse IgG (H+L), SuperclonalTM Recombinant Secondary Antibody, Alexa FluorTM 555 (Cat A28108, Lot2504690; ThermoFisher, USA)

Validation

http://www.biosupplies.com.au/assets/files/docs/400-2.pdf
SPECIFICITY
This antibody recognizes linear (1→3)-β- oligosaccharide segments in (1→3)-β-glucans. It has no cross reactivity with (1→4)-β-glucan or (1→3,1→4)-β-glucan.

Citations for Cat. #400-2: (1→3)-β-glucan-directed monoclonal antibody
Meikle, PJ, Bonig, I, Hoogenraad, NJ, Clarke, AE, Stone, BA (1991). The location of (1-3)- beta-glucans in the walls of pollen tubes of Nicotiana alata using a (1-3)-beta-glucan-specific monoclonal antibody. Planta 185 (1), 1-8.
Salnikov, VV, Grimson, MJ, Seagull, RW, et al. (2003). Localization of sucrose synthase and callose in freeze-substituted secondary- wall-stage cotton fibers. Protoplasma 221 (3-4), 175- 184.

Chiovitti, A, Higgins, MJ, Harper, RE, et al. (2003). The complex polysaccharides of the raphid diatom Pinnularia viridis (Bacillariophyceae). Journal of Phycology 39 (3), 543-554.
Ligrone, R, Vaughn, KC, Renzaglia, KS, et al. (2002). Diversity in the distribution of polysaccharide and glycoprotein epitopes in the cell walls of bryophytes: new evidence for the multiple evolution of water-conducting cells. New Phytologist 156 (3), 491-508.

Mogami, N, Shiota, H, Tanaka, I (2002). LP28, a lily pollen-specific LEA-like protein, is located in the callosic cell wall during male gametogenesis. Sexual Plant Reproduction 15 (2), 57-63.
Serpe, MD, Muir, AJ, Driouich, A (2002). Immunolocalization of beta-D-glucans, pectins, and arabinogalactan-proteins during intrusive growth and elongation of nonarticulated laticifers in Asclepias speciosa Torr. Planta 215 (3), 357-370.

Trethewey, JAK, Harris, PJ (2002). Location of (1-3)- and (1-3),(1-4)-beta-D-glucans in vegetative cell walls of barley (Hordeum vulgare) using immunogold labeling. New Phytologist 154 (2), 347-358.
Park, SK, Twell, D (2001). Novel patterns of ectopic cell plate growth and lipid body distribution in the Arabidopsis gemini pollen1 mutant. Plant Physiology 126 (2), 899-909.

Humbel, BM, Konomi, M, Takagi, T, et al. (2001). In situ localization of beta-glucans in the cell wall of Schizosaccharomyces pombe. Yeast 18 (5), 433-444.
Dahiya, P, Brewin, NJ (2000). Immunogold localization of callose and other cell wall components in pea nodule transfer cells. Protoplasma 214 (3-4), 210-218.

Sivaguru, M, Fujiwara, T, Samaj, J, et al. (2000). Aluminum-induced 1-3-beta-D-glucan inhibits cell-to-cell trafficking of molecules through plasmodesmata. A new mechanism of aluminum toxicity in plants. Plant Physiology 124 (3), 991-1005.
Hasegawa, Y, Nakamura, S, Uheda, E, et al. (2000). Immunolocalization and possible roles of pectins during pollen growth and callose plug formation in angiosperms Grana 39 (1), 46- 55.

Otegui, M, Staehelin, LA (2000). Syncytial-type cell plates: A novel kind of cell plate involved in endosperm cellularization of Arabidopsis. Plant Cell 12 (6), 933-947.
Orfila, C, Knox, JP, Ferguson, C, Teeri, TT, Siika-aho, M, et al. (1998). Location of cellulose and callose in pollen tubes and grains of Nicotiana tabacum. Planta 206 (3), 452-460.

Enkerli, K, Hahn, MG, Mims, CW (1997). Immunogold localization of callose and other plant cell wall components in soybean roots infected with the oomycete Phytophthora sojae. Canadian Journal of Botany 75 (9), 1509-1517.
Kudlicka, K, Brown, RM (1997). Cellulose and callose biosynthesis in higher plants .1. Solubilization and separation of (1-3)- and (1-4)- beta-glucan synthase activities from mung bean. Plant Physiology 115 (2), 643-656.

Lemoine, MC, Gollotte, A, Gianinazzipearson, V. (1995). Localisation of beta-(1-3)-glucan in walls of the endomycorrhizal fungi Glomus mosseae (Nicol and Gerd) Gerd and Trappe and Acaulospora laevis Gerd and Trappe during colonization of host roots. New Phytologist 129 (1), 97-105
Gianinazzipearson, V, Lemoine, MC, Arnould, C, et al. (1994). Localisation of beta-(1-3) glucans in spore and hyphal walls of fungi in the Glomales. Mycologia 86 (4), 478-485.

Lin J, Wester MJ, Graus MS, Lidke KA, Neumann AK (2016). Nanoscopic cell-wall architecture of an immunogenic ligand in Candida albicans during antifungal drug treatment. Mol Biol Cell. 27(6):1002-14
Pappas HC, Sylejmani R, Graus MS, Donabedian PL, Whitten DG, Neumann AK (2016). Antifungal Properties of Cationic Phenylene Ethynylenes and Their Impact on β-Glucan Exposure. Antimicrob Agents Chemother. 60(8):4519-29

Hopke A, Nicke N, Hidu EE, Degani G, Popolo L, Wheeler RT (2016). Neutrophil Attack Triggers Extracellular Trap-Dependent Candida Cell Wall Remodeling and Altered Immune Recognition. PLoS Pathog. 12(5):e1005644
Zhang SQ, Zou Z, Shen H, Shen SS, Miao Q, Huang X, Liu W, Li LP, Chen SM, Yan L, Zhang JD, Zhao JJ, Xu GT, An MM, Jiang YY (2016). Mnn10 Maintains Pathogenicity in Candida albicans by Extending α-1,6-Mannose Backbone to Evade Host Dectin-1 Mediated Antifungal Immunity. PLoS Pathog. 12(5):e1005617

Shen H, Chen SM, Liu W, Zhu F, He LJ, Zhang JD, Zhang SQ, Yan L, Xu Z, Xu GT, An MM, Jiang YY (2015). Abolishing Cell Wall Glycosylphosphatidylinositol-Anchored Proteins in Candida albicans Enhances Recognition by Host Dectin-1. Infect Immun. 83(7):2694-704
Davis SE, Hopke A, Minkin SC Jr, Montedonico AE, Wheeler RT, Reynolds TB (2014) .Masking of β(1-3)-glucan in the cell wall of Candida albicans from detection by innate immune cells depends on phosphatidylserine. Infect Immun. 82(10):4405-13

Marakalala MJ, Vautier S, Potrykus J, Walker LA, Shepardson KM, Hopke A, Mora-Montes HM, Kerrigan A, Netea MG, Murray GI, Maccallum DM, Wheeler R, Munro CA, Gow NA, Cramer RA, Brown AJ, Brown GD (2013). Differential adaptation of Candida albicans in vivo modulates immune recognition by dectin-1. PLoS Pathog. 9(4):e1003315

McLellan CA, Whitesell L, King OD, Lancaster AK, Mazitschek R, Lindquist S (2012). Inhibiting GPI anchor biosynthesis in fungi stresses the endoplasmic reticulum and enhances immunogenicity. ACS Chem Biol. 7(9):1520-8
Klippel N, Cui S, Groebe L, Bilitewski U (2010). Deletion of the Candida albicans histidine kinase gene CHK1 improves recognition by phagocytes through an increased exposure of cell wall beta-1,3-glucans. Microbiology. 156(Pt 11):3432-44

Wheeler RT, Kombe D, Agarwala SD, Fink GR (2008). Dynamic, morphotype-specific Candida albicans beta-glucan exposure during infection and drug treatment. PLoS Pathog. 4(12)

Wheeler RT, Fink GR (2006). A drug-sensitive genetic network masks fungi from the immune system. PLoS Pathog. 2(4):e35.

This checklist template is licensed under a Creative Commons Attribution 4.0 International License, which permits use, sharing, adaptation, distribution and reproduction in any medium or format, as long as you give appropriate credit to the original author(s) and the source, provide a link to the Creative Commons license, and indicate if changes were made. The images or other third party material in this article are included in the article's Creative Commons license, unless indicated otherwise in a credit line to the material. If material is not included in the article's Creative Commons license and your intended use is not permitted by statutory regulation or exceeds the permitted use, you will need to obtain permission directly from the copyright holder. To view a copy of this license, visit http://creativecommons.org/licenses/by/4.0/
